# Structural and functional insights into the candidate genes associated with different developmental stages of flag leaf in bread wheat (*Triticum aestivum* L.)

**DOI:** 10.3389/fgene.2022.933560

**Published:** 2022-08-24

**Authors:** Sheetal Mehla, Upendra Kumar, Prexha Kapoor, Yogita Singh, Pooja Sihag, Vijeta Sagwal, Priyanka Balyan, Anuj Kumar, Navjeet Ahalawat, Nita Lakra, Krishna Pal Singh, Vladan Pesic, Ivica Djalovic, Reyazul Rouf Mir, Om Parkash Dhankher

**Affiliations:** ^1^ Department of Molecular Biology, Biotechnology and Bioinformatics, College of Biotechnology, CCS Haryana Agricultural University, Hisar, India; ^2^ Department of Botany, Deva Nagri P. G. College, CCS University, Meerut, India; ^3^ Shantou University Medical College, Shantou, China; ^4^ Dalhousie University, Halifax, NS, Canada; ^5^ Biophysics Unit, College of Basic Sciences and Humanities, GB Pant University of Agriculture and Technology, Pantnagar, India; ^6^ Vice-Chancellor’s Secretariat, Mahatma Jyotiba Phule Rohilkhand University, Bareilly, India; ^7^ Department of Genetics and Plant Breeding, Faculty of Agriculture, University of Belgrade, Belgrade, Serbia; ^8^ Institute of Field and Vegetable Crops, National Institute of the Republic of Serbia, Maxim Gorki 30, Novi Sad, Serbia; ^9^ Division of Genetics and Plant Breeding, Sher-e-Kashmir University of Agricultural Sciences and Technology of Kashmir (SKUAST-Kashmir), Srinagar, India; ^10^ Stockbridge School of Agriculture, University of Massachusetts Amherst, Amherst, MA, United States

**Keywords:** flag leaf development, homology modeling, gene expression analysis, qRT-PCR (quantitative real-time polymerase chain reaction), MD simulation

## Abstract

Grain yield is one of the most important aims for combating the needs of the growing world population. The role of development and nutrient transfer in flag leaf for higher yields at the grain level is well known. It is a great challenge to properly exploit this knowledge because all the processes, starting from the emergence of the flag leaf to the grain filling stages of wheat (*Triticum aestivum* L.), are very complex biochemical and physiological processes to address. This study was conducted with the primary goal of functionally and structurally annotating the candidate genes associated with different developmental stages of flag leaf in a comprehensive manner using a plethora of *in silico* tools. Flag leaf-associated genes were analyzed for their structural and functional impacts using a set of bioinformatics tools and algorithms. The results revealed the association of 17 candidate genes with different stages of flag leaf development in wheat crop. Of these 17 candidate genes, the expression analysis results revealed the upregulation of genes such as *TaSRT1-5D*, *TaPNH1-7B*, and *TaNfl1-2B* and the downregulation of genes such as *TaNAP1-7B*, *TaNOL-4D*, and *TaOsl2-2B* can be utilized for the generation of high-yielding wheat varieties. Through MD simulation and other *in silico* analyses, all these proteins were found to be stable. Based on the outcome of bioinformatics and molecular analysis, the identified candidate genes were found to play principal roles in the flag leaf development process and can be utilized for higher-yield wheat production.

## Graphical Abstract

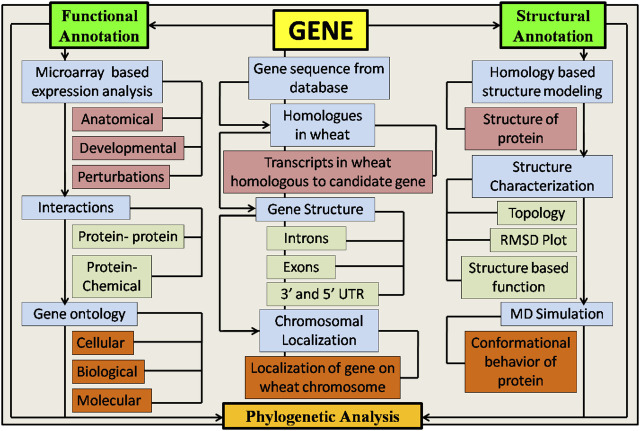

## 1 Introduction

Bread wheat (*Triticum aestivum* L.) has been considered one of the initial food crops that have been domesticated as a staple food for thousands of years in the major civilizations of West Asia, North America, and Europe (Giraldo et al., 2019). The area under the cultivation of wheat is 239.63 million hectares in the world and 29.31 million hectares in India, with a total production of 899.37 million metric tonnes in the world and 103.59 million metric tonnes in India (FAO, 2020). The cereal utilization forecast for the years 2020–21 has been raised to 766 million tonnes on a global scale, 54 million tonnes (2%) above the 2019–20 level and 4.3 million tonnes higher than previous reports^1^. The grain yield in wheat is driven by the amount of energy harvested by upper leaves. Hence, it is said that wheat is a limited-source crop. Approximately 75% of the overall yield is contributed by the flag leaf and spike in wheat^2^. As evident from the previous reports, the supply of wheat needs to be increased by one billion metric tonnes to meet the needs of an increasing population. All parts of the plant contribute to the development of spikes in cereal crops (Blade and Baker, 1991). However, it was discovered that the uppermost three leaves determine cereal yield potential due to their importance in grain filling (Bi̇rsi̇n, 2005). It was found that the defoliation of flag leaf generated an 18–30% loss in grain yield in wheat (Youssef and Salem, 1976; Banitaba et al., 2007). Therefore, it is clear that studying the molecular pathways of flag leaf development is critical for gaining a better understanding of its function and meeting the needs of its exploration to combat the need for food supply.

Photosynthesis is the ultimate process for increasing grain yield in wheat, and the flag leaf is the part of the plant that receives the highest amount of sunlight for the preparation of food. A flag leaf is referred to as the last leaf to emerge from indeterminate plants of the Poaceae family (Palmer et al., 2015). In cereals, the flag leaf is known to provide the largest portion of photoassimilates for grain filling (Biswal and Kohli, 2013). Wheat is known to be a source-limited crop, as its yield is primarily driven through the amount of energy captured by the upper leaves (Koshkin and Tararina, 1989). Morphological characteristics such as the shape and area of the flag leaf contribute to wheat productivity (Liu K. et al., 2018). Yield-related traits were found to be positively associated with the size of flag leaf in wheat (Fan et al., 2015; Liu K. et al., 2018, Liu et al., 2018 Y.). Duwayri (1984) reported that the removal of flag leaves resulted in reduced grain yield and kernel numbers. Different stages occur during the development of a flag leaf, each with its specific function. Flag leaves provide the storage organs with photosynthetic products (Yan et al., 2020); hence, it becomes necessary to identify candidate genes associated with different stages of flag leaf development. The synergy of leaf development and environmental factors results in the complex trait of natural senescence (Liu et al., 2016).

Despite the significance of flag leaves in wheat production, a lesser amount of information about their molecular, morphological, and physiological characteristics is available. In the present study, the main objective was to initially identify and then characterize the genes associated with flag leaf development and perform a detailed analysis of proteins encoded in wheat. Through computational analysis, 17 candidate genes were found to be involved in flag leaf development from reference sequences of the wheat genome. These genes were further structurally and functionally annotated through various *in silico* tools and automated servers. Structural annotation involved the elucidation of gene structure and protein modeling followed by molecular dynamics simulation configuration at different nanoseconds and their physiochemical properties, whereas functional annotation involved gene ontology, phylogenetic relationships, identification of functional domains, molecular interaction networks, and microarray profiling.

## 2 Materials and methods

### 2.1 Structural annotation

#### 2.1.1 Identification and chromosomal localization of candidate genes involved in wheat flag leaf development

The protein sequences of candidate genes associated with the development of flag leaf were retrieved from different model plants such as *Arabidopsis thaliana* and *Oryza sativa* using the NCBI database (www.ncbi.nlm.nih.gov) (Jenuth, 2000). The orthologs of these proteins responsible for flag leaf development in wheat were identified by performing BLASTp (http://plants.ensembl.org/Triticum_aestivum/Tools/Blast) against the Ensembl Plants database (Bolser et al., 2015) with a selection of *T. aestivum,* which contains data from different assemblies, namely, PGSBv2.0, Elv1.1, and WeebilV1. The genes associated with these predicted proteins were further annotated for their chromosomal localization in the wheat genome (Kumar et al., 2018b).

#### 2.1.2 Identification of gene structure

The Gene Structure Display 2.0 (GSDV2) (http://gsds.gao-lab.org/) server was utilized to explore information regarding the detailed structure of genes. The coding and genomic sequences of the predicted proteins were taken into account for marking three major regions of a gene: 3′ and 5′ UTRs, exons, and introns.

#### 2.1.3 Physiochemical properties of candidate proteins

Physiochemical properties are the basic properties of a particular protein, such as the theoretical isoelectric point, instability index, molecular weight, and aliphatic index. The ProtParam server (https://web.expasy.org/protparam/) (Garg et al., 2016) was used to analyze all these parameters of candidate proteins associated with flag leaf development in the wheat crop.

#### 2.1.4 Homology modeling

3D structural modeling of all 17 candidate proteins associated with the development of flag leaf was performed through the SWISS-MODEL Server (https://swissmodel.expasy.org/) based on the homology approach. Protein sequences were placed in the target sequence dialog box and a template search was initiated. A template was selected from a set of templates ranging from 3 to 50 concerning PSI BLAST and HHblits. Based on the similarity, the most suitable template was selected for performing homology-based modeling of candidate genes. The structures of proteins after modeling were further analyzed with UCSF ChimeraX software (https://www.cgl.ucsf.edu/chimerax/) and the ProFunc server (http://www.ebi.ac.uk/thornton-srv/databases/ProFunc/) for proper characterization both at the surface and internal levels. The quality of the protein was checked through the PROCHECK server by dihedral analysis of the Ramachandran plots of predicted candidate proteins.

### 2.2 Annotating candidate genes at the functional level

Functional annotation is the process which includes the annotation of a particular biological function to biomolecules based on a computational approach by comparing the query data with the existing datasets selected in the database. This is one of the most prominent steps after structural annotation of biomolecules (Humann et al., 2019). Functional annotation was performed by analyzing the gene ontology analysis, expression profiling, and molecular simulation studies of the candidate proteins.

#### 2.2.1 Gene ontology

Complete functional annotation of the 17 candidate genes was performed at both the nucleotide and protein levels. At the cellular, molecular, and biological levels, gene ontology at the nucleotide level of the candidate genes was performed using the Gene Ontology Resource (http://geneontology.org), Ensembl database (https://plants.ensembl.org/index.html), URGI (https://wheat-urgi.versailles.inra.fr/), and UniParc (https://www.uniprot.org/help/uniparc). For the gene ontology study at the structural level, the modeled protein structures were analyzed with the help of the automated ProFunc server (http://www.ebi.ac.uk/thornton-srv/databases/ProFunc/) for the proper depiction of functions at the cellular, biological, and biochemical levels.

#### 2.2.2 Gene expression profiling

The expression profiling of all the candidate genes was performed with the help of GENEVESTIGATOR (https://genevestigator.com/) at different levels. First, at the anatomical level, depiction was performed on a quantitative basis in different tissues of wheat, taking into account the TA_mRNASeq_WHEAT_GL-0 dataset and selecting all 17 candidate genes in a heat map format. Second, at ten different developmental stages, a set of experiments was selected as the desired perturbation for the presence of 17 candidate genes in a heat map format.

#### 2.2.3 Experimental validation of candidate genes through expression analysis

##### 2.2.3.1 Plant material and sampling stages

**FIGURE 1:**
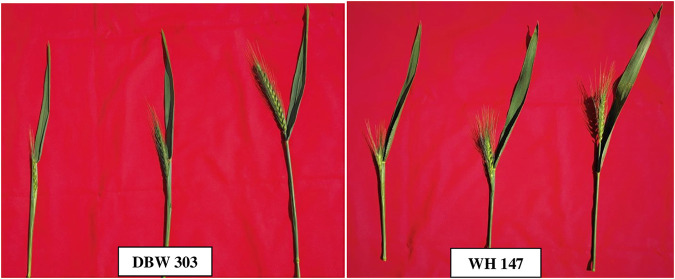
Morphology of the flag leaf of DBW303 and WH147 at different stages of sample collection.

For experimental validation of candidate genes, two varieties DBW303 (high yielding) and WH147 (low yielding) were selected. The plants were grown at the research field of wheat and barley section, the Department of Genetics and Plant Breeding, Chaudhary Charan Singh Haryana Agricultural University, Hisar, India. The flag leaf samples of the varieties were collected at three different developmental stages: flag leaf emergence, fully developed flag leaf, and flag leaf senescence (Figure 1).

##### 2.2.3.2 Relative expression of candidate genes in high- and low-yielding wheat varieties

Total RNA was isolated from 50 to 100 mg flag leaf tissue of selected varieties using a Maxwell RSC Plant RNA Kit (Promega, United States) according to the manufacturer’s recommendations. A RevertAid cDNA Synthesis Kit (Thermo Scientific, United States) was used for the synthesis of first-strand cDNA from the total RNA isolated. The primers of all the 17 genes along with actin as an endogenous control were designed with the help of Primer Express Software v3.0.1 (Applied Biosystems). A detailed list of the primers used is given in Supplementary Table S1. Quantitative real-time polymerase chain reaction was performed using a QuantaStudio^TM^ 6 Flex Real-Time PCR Detection System (Applied Biosystems) with the PowerUp^TM^ SYBR^TM^ Green PCR Master Mix (Applied Biosystem) for 2 min at 50°C for UDG activation, 10 min at 95°C for initial denaturation followed by 40 cycles of 15 s of denaturation at 95°C, 15 s of annealing 53°C and extension at 72°C for 1 min). Fold changes were calculated by the 2^-∆∆Ct^ method.

#### 2.2.4 Network analysis of candidate genes with native protein and chemical interactors

Protein–protein and protein–chemical interaction network analyses were performed with the help of servers such as GeneMANIA (https://genemania.org/) and the STITCH v 5.0 (http://stitch.embl.de/), respectively. Network analysis was performed to identify the biomolecules interacting with the candidate genes for proper functional annotation of genes encoding proteins.

##### 2.2.5 Molecular dynamics simulation

To evaluate the stability of the predicted 3D structures, MD simulations were performed. Modeled 3D structures from the SWISS-MODEL server were used as the initial configuration of all simulations. The MD simulations were conducted as per the protocol previously described in Gajula et al. (2016); Kumar et al. (2016, 2018a, and 2018b); Mathpal et al. (2018); and Gautam et al. (2019). A minimum of 10 Å distance was used between the protein surfaces and the simulation box edges. All the systems were solvated with the TIP3P water model. Cl^−^ and Na^+^ were further added to the simulation boxes to neutralize the systems. All unbiased MD simulations were carried out using GROMACS-2020 (https://www.gromacs.org) with an all-atom CHARMM36m force field. All the systems were energy minimized and equilibrated with different initial atomic velocities using the following steps: 1)minimization of energy to steepest descent level, 2) restrainment of position (all heavy atoms of protein) NVT [moles (N), volume (V), and temperature (T)] simulation with a restraining force constant of 1,000 kJ/mol, and 3) a 500 ps position-restrained NPT [moles (N), pressure (P), and temperature (T)] simulation with a restraining force constant of 1,000 kJ/mol. The NPT ensemble was utilized for the simulation production at 20 ns with an average temperature of 300 K *via* the v-rescale thermostat and Parrinello–Rahman barostat with 2.0 ps as the coupling constant. For the cutoff of Lenard-Jones and other short-range electrostatic interactions, a Verlet cutoff scheme was employed with a cutoff of 1.0 nm throughout the simulation. The particle mesh Ewald summation method was employed to treat electrostatic interactions over a long range. The LINCS algorithm was used for the constraint of all hydrogen atoms, and the SETTLE algorithm was used for constraining all the bonds and angles of TIP3P water molecules. The root means square deviation (RMSD) of Cα atoms of protein concerning a reference structure was calculated with the GROMACS program “gmxrms” by least-square fitting the structure to the reference structure. Principal component analysis (PCA) is a widely used technique to highlight the slowest functional motions of proteins (Bahar et al., 1998). The principal components (PCs) representing the collective motion of the proteins were obtained after diagonalization of the covariance matrix. The eigenvectors and eigenvalues of the covariance matrix represent the principal direction of the motion and the magnitude of motion along with it, respectively. The GROMACS tools “gmxcovar” and “gmxanaeig” were used for the calculation of the covariance matrix and PC projections.

### 2.3 Phylogenetic analysis

Phylogenetic analysis was conducted to obtain information regarding the relatedness among the 17 candidate proteins, and the phylogenetic tree was constructed with the help of Mega X (https://www.megasoftware.net/) software using the neighbor-end joining method with 1,000 bootstraps.

## 3 Results

### 3.1 Identification and chromosomal localization of candidate genes in the wheat genome associated with flag leaf development

**FIGURE 2:**
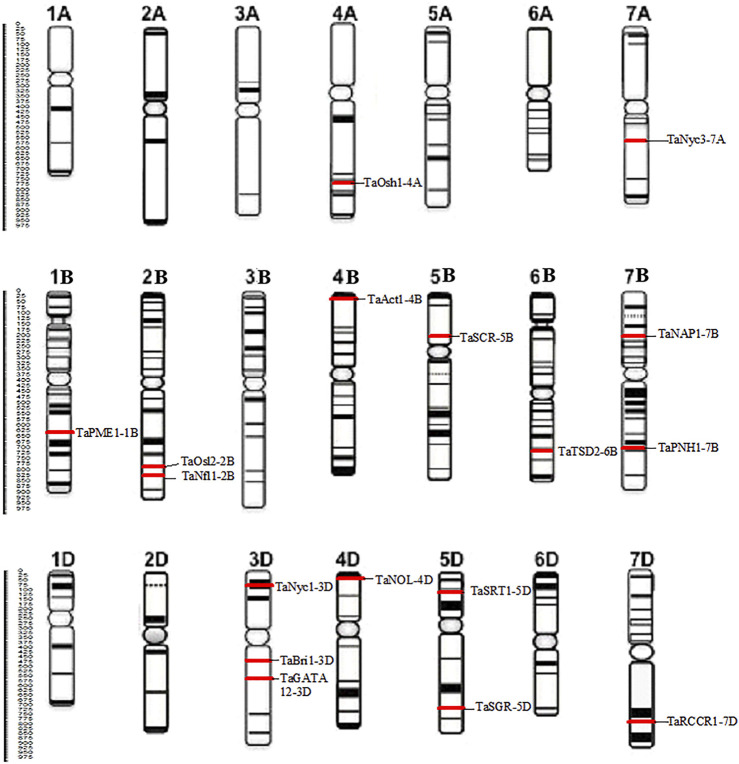
Distribution of 17 candidate genes associated with the development of the flag leaf on different chromosomes of wheat.

The protein sequences of candidate genes associated with the development of flag leaf were retrieved from different plants such as *Oryza sativa* and *Arabidopsis thaliana,* and the orthologs of these proteins responsible for flag leaf development in wheat were identified by BLASTp. All 17 candidate genes that were identified from the Ensembl Plants database were annotated with the help of various bioinformatics tools. The BLASTp results yielded more than 50 wheat scaffolds, of which the one with the highest sequence identity with the respective genes was taken as an ortholog for further analysis (Dhaliwal et al., 2014).With each gene queried, numerous associated transcripts were found. The full-length transcripts of candidate genes related to flag leaf genes were found to have 67.6–99.4% identity with the query sequence. The size of the CDS ranged from 1,428 to 4,445 bp, and the subsequent protein length ranged from 280 to 1,124 amino acids (Table 1). In total, 17 flag leaf-associated genes were found to be scattered on chromosomes 1B, 2B, 3D, 4A, 4B, 4D, 5B, 5D, 6B, 7A, 7B, and 7D of wheat (Figure 2).

**TABLE 1:** List of identified flag leaf development–associated genes along with their basic characteristics elucidated with the help of the Ensembl Plants database.

S No.	Gene	Ensembl ID	Location	Cellular location	CDS	AA
1	*TaAct1-4B*	TraesCS4B02G050600	4B	Cytoskeleton	1982	385
2	*TaBri1-3D*	TraesCS3D02G246500	3D	Endosome, membrane	4,445	1,124
3	*TaGATA12-3D*	TraesCS3D02G274600	3D	Nucleus	1747	386
4	*TaNAP1-7B*	TraesCS7B02G167200	7B	Cytoplasm	4,074	1,357
5	*TaNfl1-2B*	TraesCS2B02G464200	2B	Nucleus	1,428	392
6	*TaNOL-4D*	TraesCS4D02G012100	4D	Chloroplast thylakoid	1,432	345
7	*TaNyc1-3D*	TraesCS3D02G159800	3D	Plastid, integral part of membrane	2,121	499
8	*TaNyc3-7A*	TraesCS7A02G338900	7A	Chloroplast	2,258	547
9	*TaOsh1-4A*	TraesCS4A02G256700	4A	Nucleus	1,616	362
10	*TaOsl2-2B*	TraesCS2B02G440400	2B	Mitochondria	1,536	511
11	*TaPME1-1B*	TraesCS1B02G274500	1B	Cytoplasm	1981	555
12	*TaPNH1-7B*	TraesCS7B02G256500	7B	Cytoplasm	3,410	956
13	*TaRCCR1-7D*	TraesCS7D02G492300	7D	Chloroplast	1,565	455
14	*TaSCR-5B*	TraesCS5B02G143100	5B	Nucleus	2,339	638
15	*TaSGR-5D*	TraesCS5D02G325900	5D	Chloroplast mitochondria	1798	280
16	*TaSRT1-5D*	TraesCS5D02G066000	5D	Nucleus	2,870	755
17	*TaTSD2-6B*	TraesCS6B02G348700	6B	Golgi apparatus	2,961	660

### 3.2 Identification of gene structure

**FIGURE 3:**
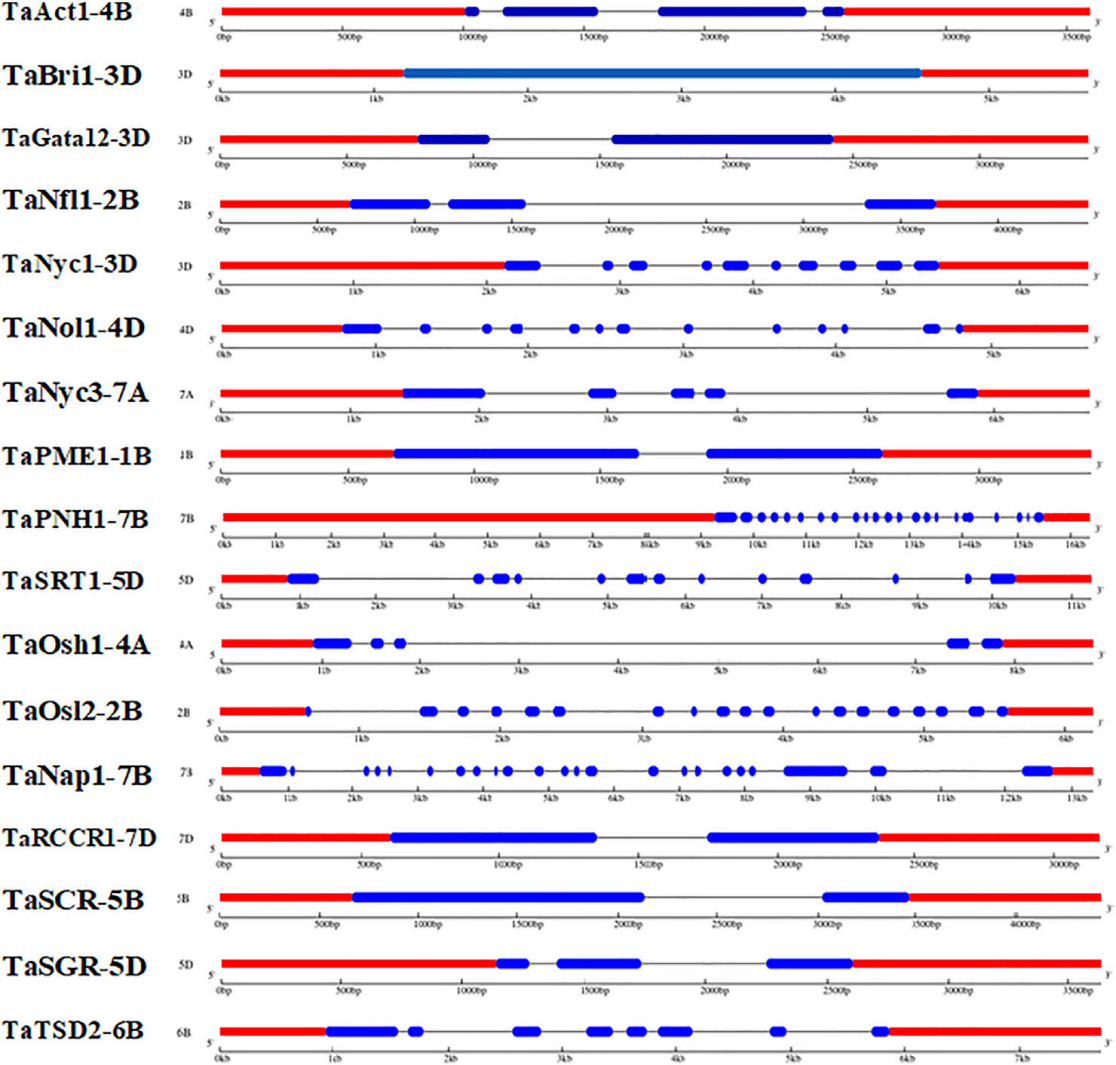
Distribution of exons and introns along with upstream and downstream regions in wheat candidate genes associated with flag leaf development (predicted by the GSDV v2.0 server).

The Gene Structure Display Server (GSDS) v 2.0 was used to elucidate the gene structures of candidate genes associated with the development of the flag leaf. The gene structure comprises exons, introns, and 3′ and 5′ untranslated regions. The number of introns ranged from one in TaGATA12-3D to 22 in TaNAP1-7B (Figure 3). The upstream and downstream regions were also analyzed for their detailed localization (Hu et al., 2015). However, the common ancestral origin of different genes was confirmed, as the exon–intron composition of flag leaf development genes was not different from their homologs.

### 3.3 Physiochemical properties of candidate proteins

The physiochemical properties of candidate proteins associated with the development of the flag leaf were elucidated using the ProtParam server. The molecular weight of translated protein ranged from 31,188.61 g/mol (TaSGR-5D) to 151,621.01 g/mol (TaNAP1-7B), and the isoelectric point (pI) ranged from 5.37 (TaAct1-4B) to 9.6 (TaNOL-4D). Out of 17 proteins, five were found to be stable in nature, while 12 were unstable, with their instability index ranging from 40.68 to 67.91 during preliminary analysis. The higher aliphatic index of proteins ranging from 67.10 (TaGATA12-3D) to 97.54 (TaBri1-3D) suggests high stability of proteins over a wider range of temperatures. The GRAVY score ranged from −0.579 (TaOsh1-4A) to 0.009 (TaPME1-1B), indicating the hydrophilic nature of the proteins (Table 2).

**TABLE 2:** Physiochemical properties of candidate proteins associated with the development of flag leaf through the ProtParam server.

S No.	Protein	Molecular weight	Theoretical isoelectric point	Instability index	Aliphatic index	GRAVY	Stability
1	TaAct1-4B	42,740.95	5.37	35.52	85.87	−0.151	Stable
2	TaBri1-3D	120,078.06	5.8	33.29	97.54	−0.014	Stable
3	TaGATA12-3D	40,961.89	5.96	67.28	67.1	−0.445	Unstable
4	TaNAP1-7B	151,621.01	6.35	48.48	94.48	−0.147	Unstable
5	TaNfl1-2B	43,014.87	8.96	49.08	71.17	−0.493	Unstable
6	TaNOL-4D	37,714.23	9.6	42.73	80.09	−0.26	Unstable
7	TaNyc1-3D	41,890.06	8.12	37.96	89.87	0.005	Stable
8	TaNyc3-7A	42,721.67	6.02	54.32	82.27	−0.212	Unstable
9	TaOsh1-4A	40,079.08	6.28	55.69	74.75	−0.579	Unstable
10	TaOsl2-2B	48,653.93	6.81	42.22	86.15	−0.066	Unstable
11	TaPME1-1B	58,739.1	7.53	34.11	79.17	0.009	Stable
12	TaPNH1-7B	107,038.12	9.32	47.07	81.5	−0.387	Unstable
13	TaRCCR1-7D	35,590.81	6.41	46.7	94.68	0	Unstable
14	TaSCR-5B	44,271.15	7.82	67.91	82.17	−0.32	Unstable
15	TaSGR-5D	31,188.61	8.69	52.31	81.61	−0.296	Unstable
16	TaSRT1-5D	82,979.01	5.62	39.97	84.82	−0.131	Stable
17	TaTSD2-6B	71,552.22	6.61	40.68	75.09	−0.372	Unstable

### 3.4 Homology modeling

**FIGURE 4:**
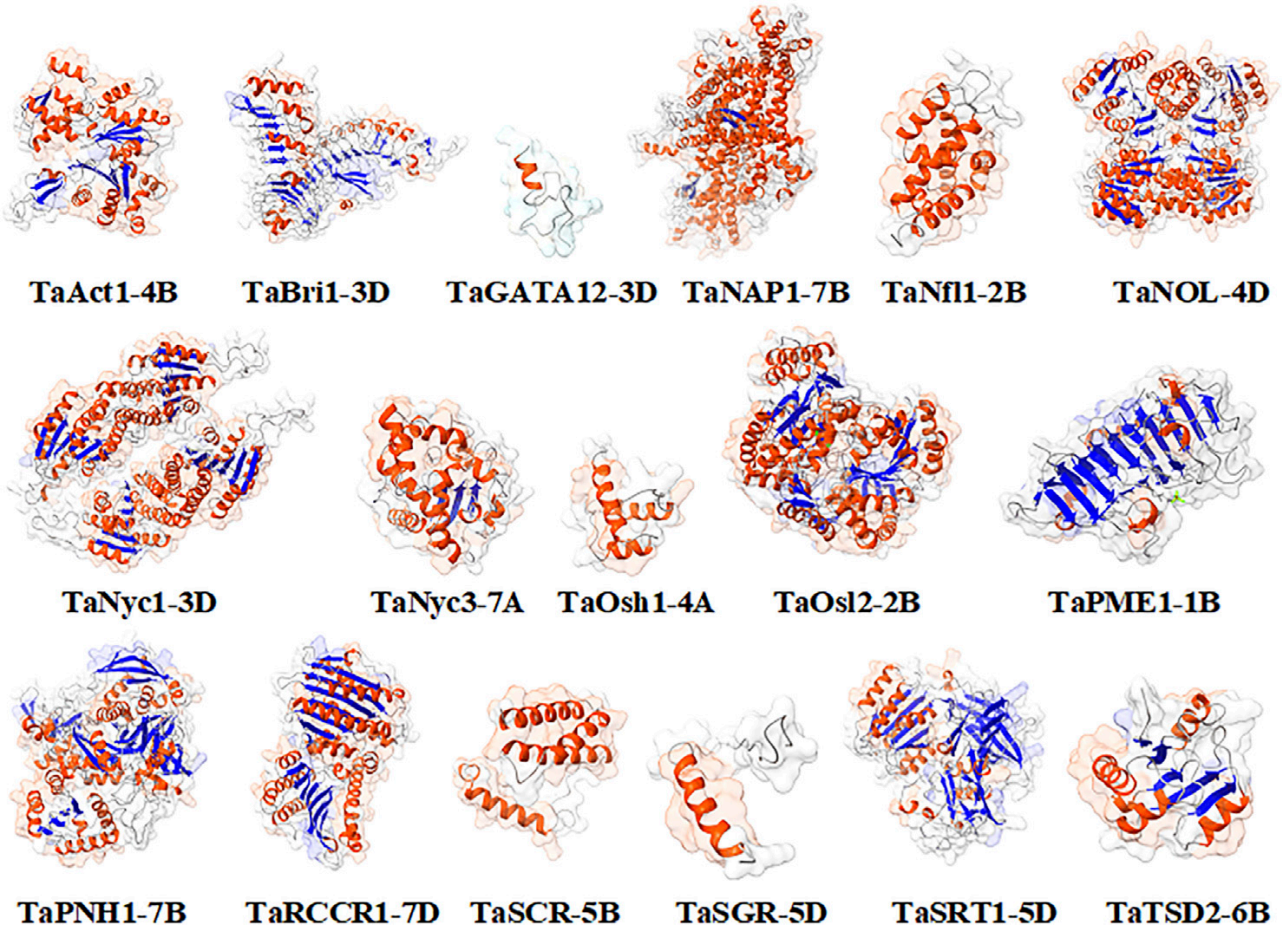
Homology-based models (molecular graphical images) produced using UCSF Chimera.

The modeling was performed based on a homology-based approach through the SWISS-MODEL server (Schwede et al., 2003; Kumar et al., 2018b; Waterhouse et al., 2018). Many templates were found ranging from 3 to 50, concerning PSI-BLAST and HHblits having a % identity ranging from 17.07 to 90.67. The state of proteins was from monomer to homodimer and homotetramer. Qualitative Model Energy Analysis (QMEAN) values of predicted proteins ranged from −0.28 in TaRCCR1-7D to −5.24 in TaNyc3-7A (Table 3). The derived structures were analyzed further with the help of UCSF ChimeraX1.1 software (Pettersen et al., 2021) for the further depiction of secondary structures such as coils, helices, sheets, and surface features (Figure 4).

**TABLE 3:** Enumeration of dihedral properties of proteins after dihedral analysis through SWISS-MODEL server.

S No.	Protein	MolProbity score	QMEAN	Stability	Solvation	Torsion	R favor (%)	State
1	TaAct1-4B	0.77	−0.48	Stable	0.01	−0.41	97.88	Monomer
2	TaBri1-3D	1.41	−1.97	Stable	−2.25	−1.35	92.05	Monomer
3	TaGATA12-3D	1.74	−2.20	Stable	0.03	−2.82	86.11	Monomer
4	TaNfl1-2B	0.87	−1.00	Stable	0.60	−1.21	97.52	Monomer
5	TaNOL-4D	2.66	−4.10	Unstable	−0.74	−3.31	89.24	Homotetramer
6	TaNyc1-3D	1.53	−3.14	Stable	−0.84	−2.61	91.88	Homotetramer
7	TaNyc3-7A	2.14	−5.24	Unstable	−0.41	−4.84	89.47	Monomer
8	TaPME1-1B	1.43	−2.88	Stable	−1.32	−2.10	93.71	Monomer
9	TaPNH1-7B	1.69	−2.02	Stable	0.08	−1.67	92.64	Monomer
10	TaSRT1-5D	1.79	−4.55	Unstable	−0.17	−4.11	89.71	Monomer
11	TaOsh1-4A	1.28	−0.90	Stable	0.61	−1.29	96.67	Monomer
12	TaOsl2-2B	1.76	−1.80	Stable	0.71	−1.87	93.79	Homodimer
13	TaNAP1-7B	1.67	−4.43	Unstable	0.26	−3.98	91.18	Monomer
14	TaRCCR1-7D	1.91	−0.28	Stable	0.68	−0.29	94.88	Homodimer
15	TaSCR-5B	1.05	−0.48	Stable	0.64	−0.87	98.04	Monomer
16	TaSGR-5D	1.45	−3.01	Stable	−3.47	−1.74	89.74	Monomer
17	TaTSD2-6B	2.41	−3.28	Stable	−2.04	−2.30	94.7	Monomer

**FIGURE 5:**
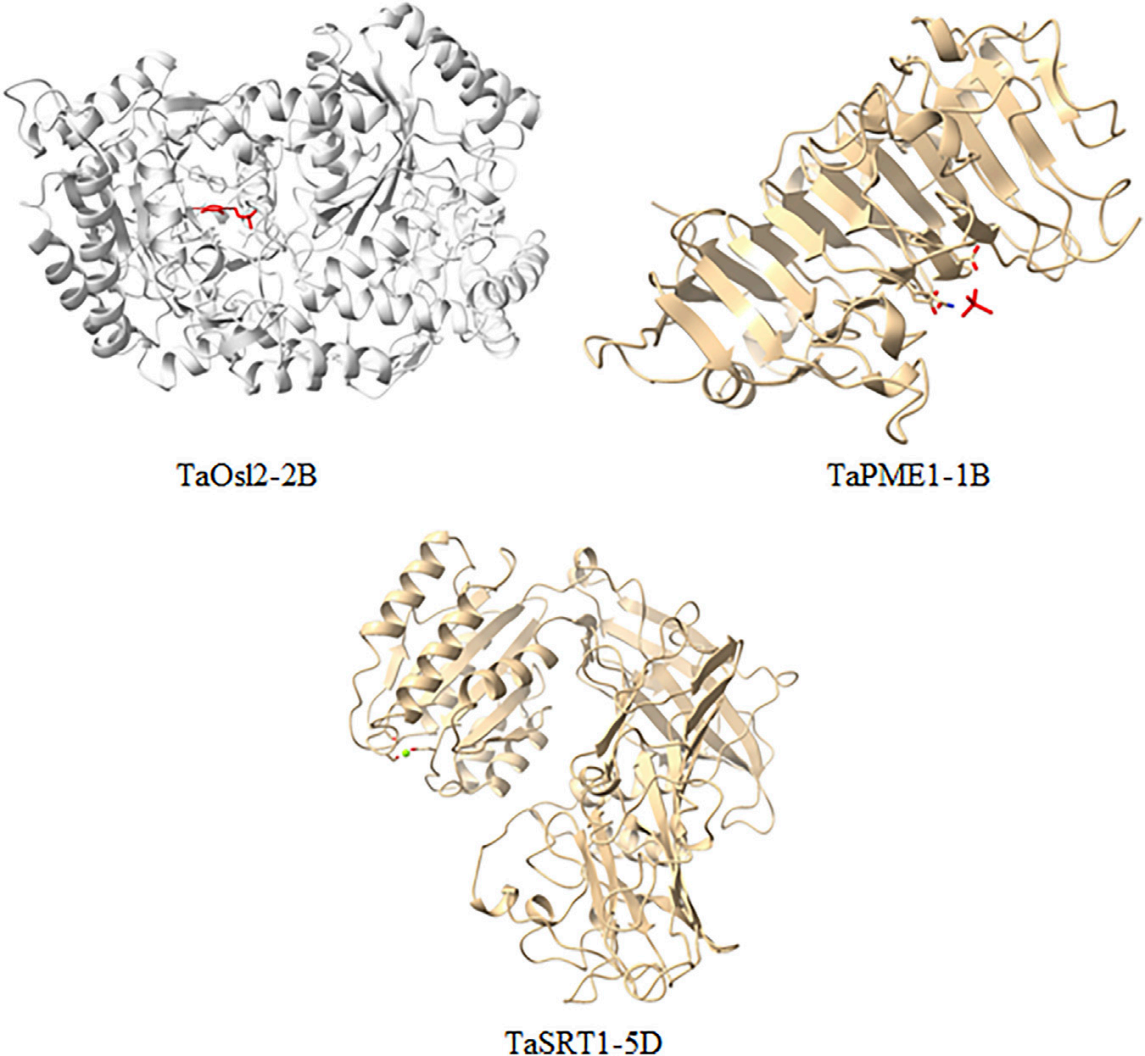
Localization of ligands interacting with the modeled protein structure through ChimeraX.

The topological architecture of proteins was predicted through the ProFunc server, which elucidates the detailed characteristics of the modeled protein structure and the ligands attached to it. Ligands such as 4′-deoxy-4′-aminopyridoxal-5′phosphate (PMP), cacodylate (CAC) ions, and magnesium (Mg) ions were found to be associated with TaOsl2-2B, TaPME1-1B, and TaSRT1-5D, respectively (Figure 5).

### 3.5 Dihedral analysis

Ramachandran dihedral statistics for modeled 3D structures of proteins associated with the development of the flag leaf in wheat deciphered with MolProbity scores ranging from 0.77 (TaAct1-4B) to 2.66 (TaNOL-4D). TaAct1-4B, TaBri1-3D, TaGATA12-3D, TaNfl-2B, TaNyc1-3D, TaPME1-1B, TaPNH1-7B, TaOsh1-4A, TaOsl2-2B, TaRCCR1-7D, TaSCR-5, TaSGR-5D, and TaTSD2-6B were all approximately zero, indicating a good agreement between the modeled structure and an experimental structure of similar size (Table 3). The PROCHECK server analysis of the modeled protein revealed a varied percentage of residues under the most favored, generously allowed, additionally allowed, and disallowed regions (Laskowski et al., 1993). The G-score that provides a measure of how normal a structure works with different proteins ranged from −0.32 (TaNyc3-7B) to 0.07 (TaNfl1-2B), indicating that the predicted models were of excellent geometry and were accepted for further analysis (Table 4; Supplementary Figure S1).

**TABLE 4:** Catalog of dihedral properties of candidate proteins elucidated through Ramachandran plot analysis.

S No.	Protein	Most favored region	Generously allowed region	Additionally allowed region	Disallowed region	G score
1	TaAct1-4B	94.2	0.0	5.80	0.0	−0.07
2	TaBri1-3D	77.2	0.7	22.1	0.0	−0.22
3	TaGATA12-3D	75.0	3.6	21.4	0.0	−0.10
4	TaNAP1-7B	89.1	1.1	9.10	0.7	−0.15
5	TaNfl1-2B	95.2	1.4	3.40	0.0	0.07
6	TaNOL-4D	89.2	1.6	9.00	0.6	−0.20
7	TaNyc1-3D	88.1	0.7	10.6	0.6	−0.13
8	TaNyc3-7A	86.1	0.5	12.5	1.0	−0.32
9	TaOsh1-4A	96.3	0.0	3.70	0.0	−0.07
10	TaOsl2-2B	87.8	0.8	10.8	0.6	−0.12
11	TaPME1-1B	87.1	0.4	12.5	0.0	−0.16
12	TaPNH1-7B	88.9	1.4	9.40	0.3	−0.09
13	TaRCCR1-7D	90.2	0.0	9.60	0.2	−0.14
14	TaSCR-5B	95.6	0.0	4.40	0.0	0.02
15	TaSGR-5D	82.4	0.0	14.7	2.9	−0.15
16	TaSRT1-5D	86.5	1.9	11.0	0.6	−0.23
17	TaTSD2-6B	88.8	1.7	8.60	0.9	−0.28

### 3.6 Functional enrichment analysis

Gene ontology enrichment analysis of genes associated with flag leaf development revealed their association with various cellular and metabolic processes. It was found that the candidate genes were involved in several biological and molecular functions, including population maintenance, leaf development, protein phosphorylation, regulation of transcription of various catabolic and anabolic processes that are required for the proper functioning of photosynthesis and nutrient transfer from flag leaf to developing grains, cellular component organization and localization, cell wall modification, and macromolecular metabolism.

### 3.7 Functional elucidation based on protein structure

The ProFunc server (Laskowski et al., 2005) was used to explore the functional annotation of genes based on protein structures derived from a homology modeling approach. It revealed the association of these modeled proteins with various cellular, biological, and biochemical processes. Cellular processes include cellular organization and localization of the cytoskeleton, periplasmic spaces, membrane, cytoplasm, and intracellular and extracellular regions. Biological processes include metabolic processes such as phosphate and pectin catabolism and carbohydrate and organic acid metabolism. Biochemical processes include the activities ofnucleotide, ATP, metal ion, and protein binding, and catalytic activities include the activities of hydrolase, oxidoreductase, nuclease, carboxylesterase, aspartyl esterase, and transaminase.

### 3.8 Profiling of gene expression

A microarray TA_mRNASeq_WHEAT_GL-0 dataset of all 17 genes was found to be available on the GENEVESTIGATOR (Grennan, 2006) platform, which was further utilized for gene expression analysis at different levels.

#### 3.8.1 Differential expression in tissues

**FIGURE 6:**
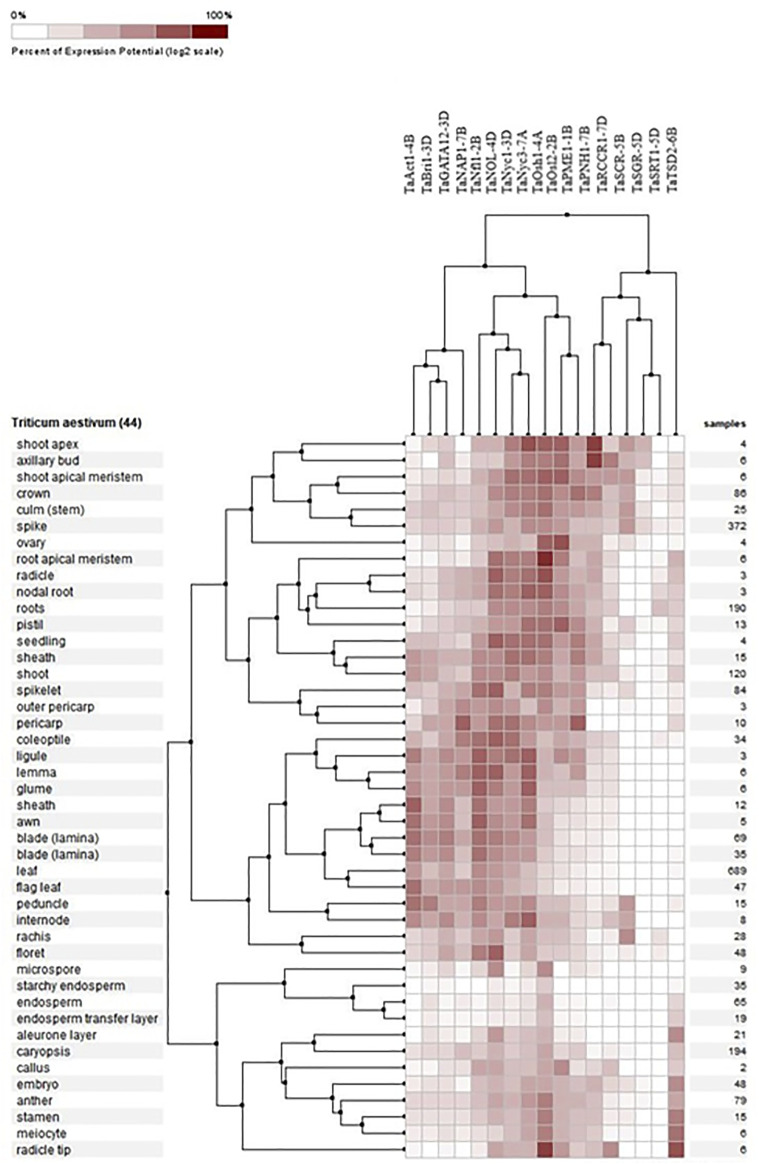
Hierarchical clustering of wheat genes based on their expression in 44 different tissues.

Expression profiling of all 17 genes revealed their presence in 44 different tissues in wheat, which were analyzed, and it was found that the expression of these genes was higher in the root tip and radicle, shoot apical meristem, seedling, and in the zone undergoing active growth, suggesting that the genes are involved in the development of tissue at the seedling stage and at later stages. TaAct1-4B was found mostly in the growing parts of the plants, especially in the flag leaf sheath and internodal areas, and it was found to be the most active gene in the flag leaf compared to the rest of the candidate genes. TaBri1-3D and TaRCCR1-7D were prominently present in the shoot apex and axillary buds (Figure 6).

#### 3.8.2 Expression of candidate genes at different developmental stages

**FIGURE 7:**
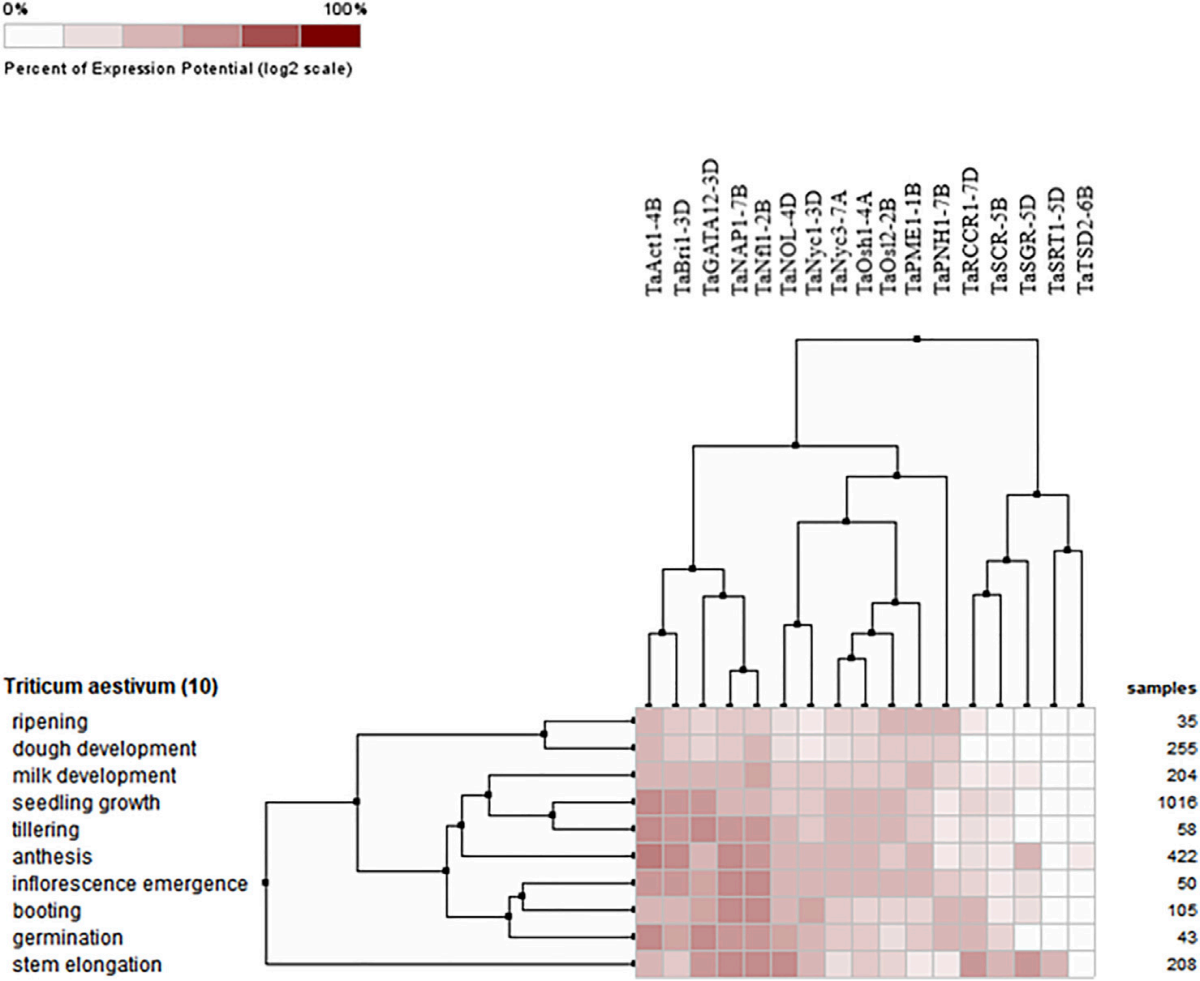
Hierarchical clustering of wheat genes based on their expression in ten different development stages.

The expression profiles of wheat genes associated with the development of flag leaf were analyzed at ten different developmental stages, including milk development, seedling growth, tillering, anthesis, inflorescence emergence, booting, germination ripening, dough development, and stem elongation. All the candidate genes either upregulated or downregulated were found to be expressed at all developmental stages. However, the expression of these genes was found to be highly prominent during germination and seed growth, deciphering their role in development; tillering, booting, and anthesis, depicting their role in the development of flag leaf, and functioning as a source–sink pathway at the time of anthesis, when the stored energy in flag leaf starts accumulating in grains (Figure 7).

#### 3.8.3 Expression during flag leaf development and senescence

##### 3.8.3.1 Flag leaf development

**FIGURE 8:**
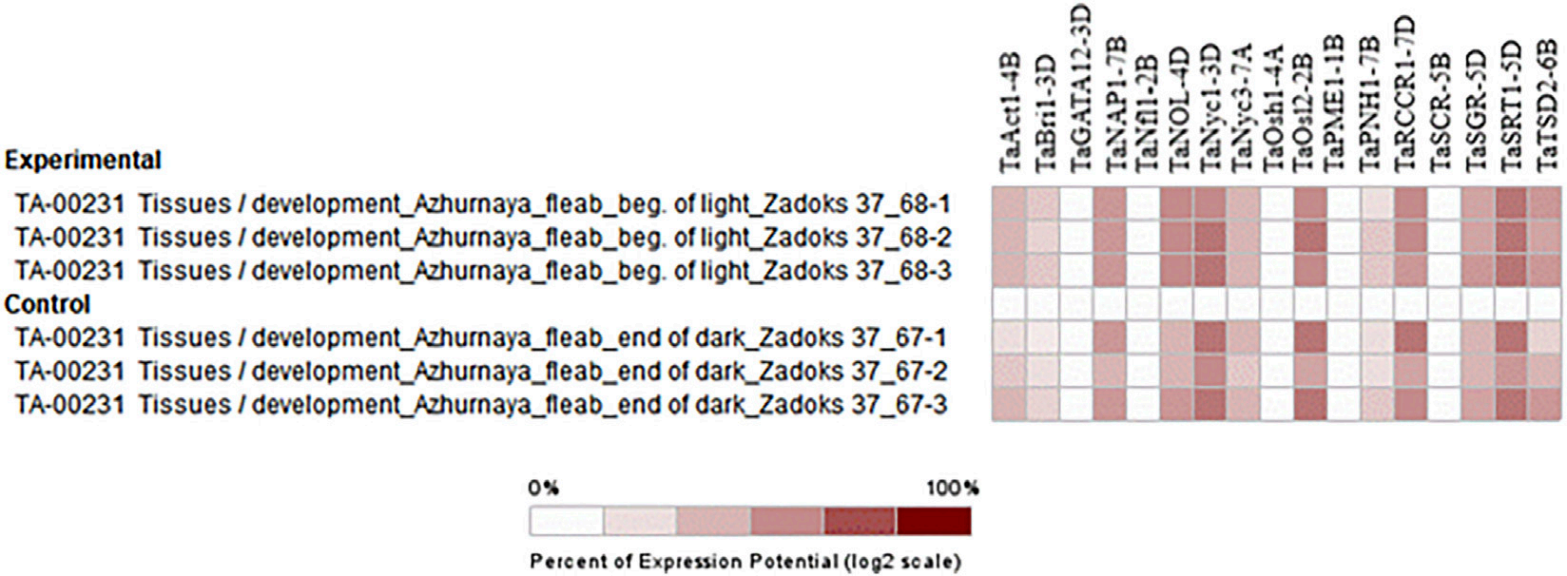
Hierarchical clustering of wheat genes based on their expression under the emergence stage in flag leaf.

Absolute expression analysis was performed by selecting perturbations where the flag leaf blade was harvested at the beginning of the light period (15 min after lights went on) from Azhurunaya plants grown to Zadoks 37 (flag leaf just visible) in a growth chamber under 16 h light/8 h dark cycles compared to 15 min before lights went on. The higher expression of genes such as *TaNAP1-7B*, *TaNOL-4D*, *TaNyc1-3D*, *TaOsl2-2B*, *TaSRT1-5D*, and *TaTSD2-6B* confirms their involvement in flag leaf development in wheat through processes such as cellular organization, leaf development, and photosynthesis (Figure 8).

##### 3.8.3.2 Flag leaf senescence

**FIGURE 9:**
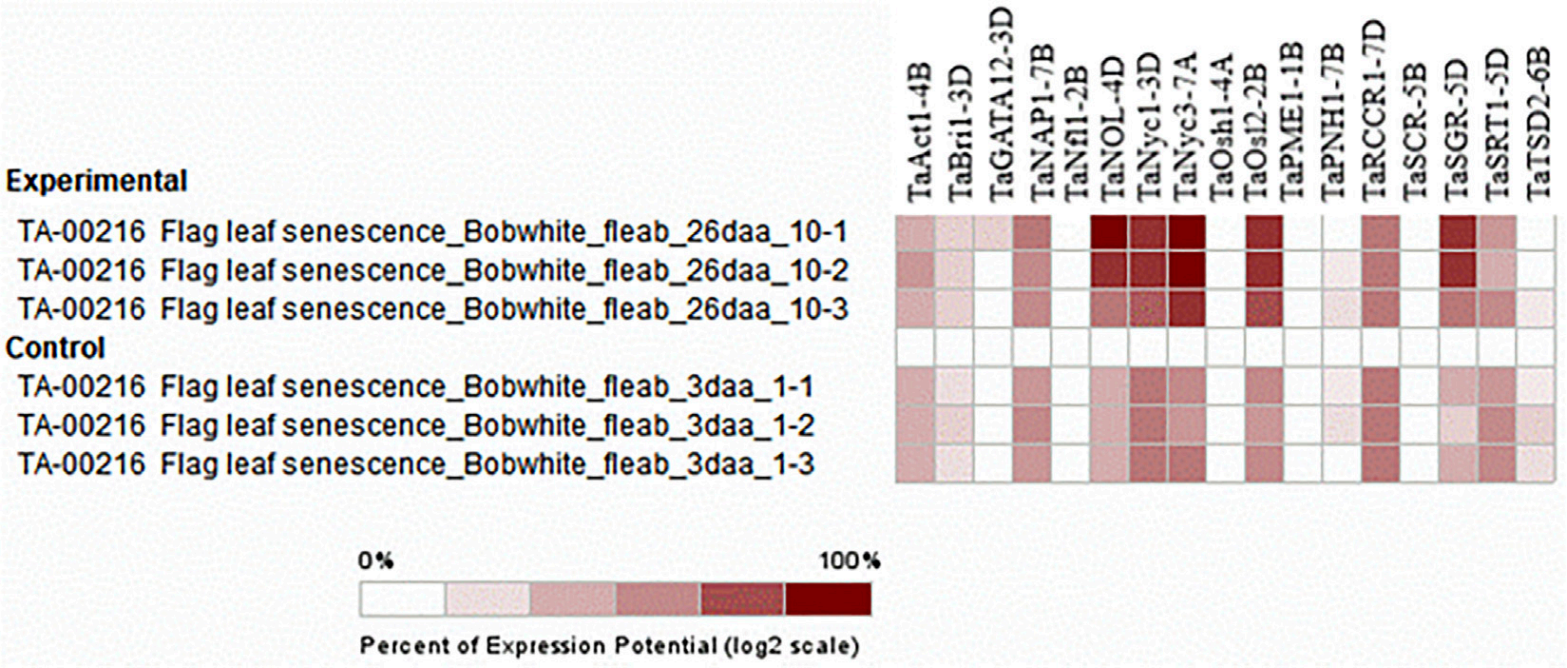
Hierarchical clustering of wheat genes based on their expression under senescence perturbations.

Selective expression analysis was performed by selecting particular perturbations involving the sampling of a 3-cm long section from the middle of the flag leaf blade 26 days after anthesis (dough development) from the main tiller of Bobwhite plants grown under 16 h light at 20°C/8 h dark at 15°C cycles in 1-L pots filled with Peters Field Cereal Mix, and 3 days after anthesis, sampling was taken as control (milk development). The chlorophyll content was ten units higher in the control sample than in the treated sample, which was 26 days after anthesis (Warburton et al., 2002). Similarly, the chlorophyll content in the flag leaf was found to increase from the time of heading to the seed setting stage and declined thereafter in cereal crops (Liu et al., 2008; Derkx et al., 2012). The biosynthesis and degradation of chlorophyll are catalyzed by a unique set of enzymes (Reinbothe et al., 2010; Hörtensteiner and Kräutler, 2011). The expression of genes associated with the degradation of chlorophyll is the first molecular indication of the onset of senescence (Hörtensteiner and Kräutler, 2011). As previously stated, the function of these genes was in metabolism, specifically the catabolism of the photosynthetic apparatus and chlorophyll, so the function of these genes can be inferred perfectly from the experiment (Figure 9).

**FIGURE 10:**
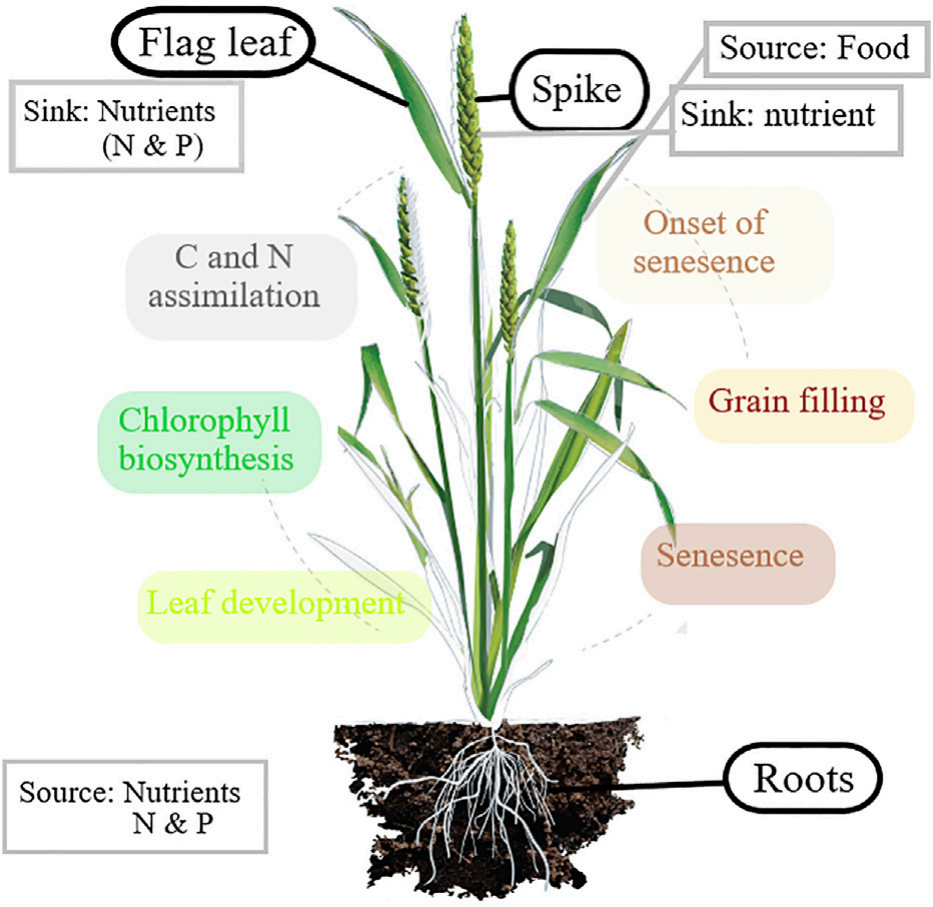
Developmental stages of flag leaf in wheat.

At the molecular level, during the development of the flag leaf, there are different phases controlled by different sets of genes. Phase 1 starts with the increase in the expression of genes involved in the development of the leaf. Phase 2 encounters an increase in the expression of genes associated with the biosynthesis of chlorophyll and other leaf functions. Phase 3 is the most active phase, involving the assimilation of carbon and nitrogen in the leaves, as mature leaves serve as a sink to store nitrogen before the anthesis phase; phase 4 begins with the onset of senescence and is characterized by the decline in leaf chlorophyll content; and upon anthesis, these leaves serve as a source of nitrogen to support the process of grain filling and utilization. Phase 5 involves the remobilization of nutrients from senescing flag leaf to the grain and other developing parts, which will ultimately lead to the complete senescence of the flag leaf. Leaf senescence is known to be an active process until death, with the main functions of recycling and reusing nutrients for the newly developing organs and enhancing the chances of survival of plants under abiotic stress (Figure 10).

### 3.9 The expression profile of candidate genes using RT-PCR

To assess the reliability and validity of *in silico* expression data and to obtain comprehensive insight into the expression profile of candidate genes in wheat in relation to high-yielding and low-yielding varieties, quantitative real-time PCR was performed using gene-specific primers for all 17 candidate genes. The fold change of the genes was analyzed relative to the other two stages of the same variety only.

**FIGURE 11:**
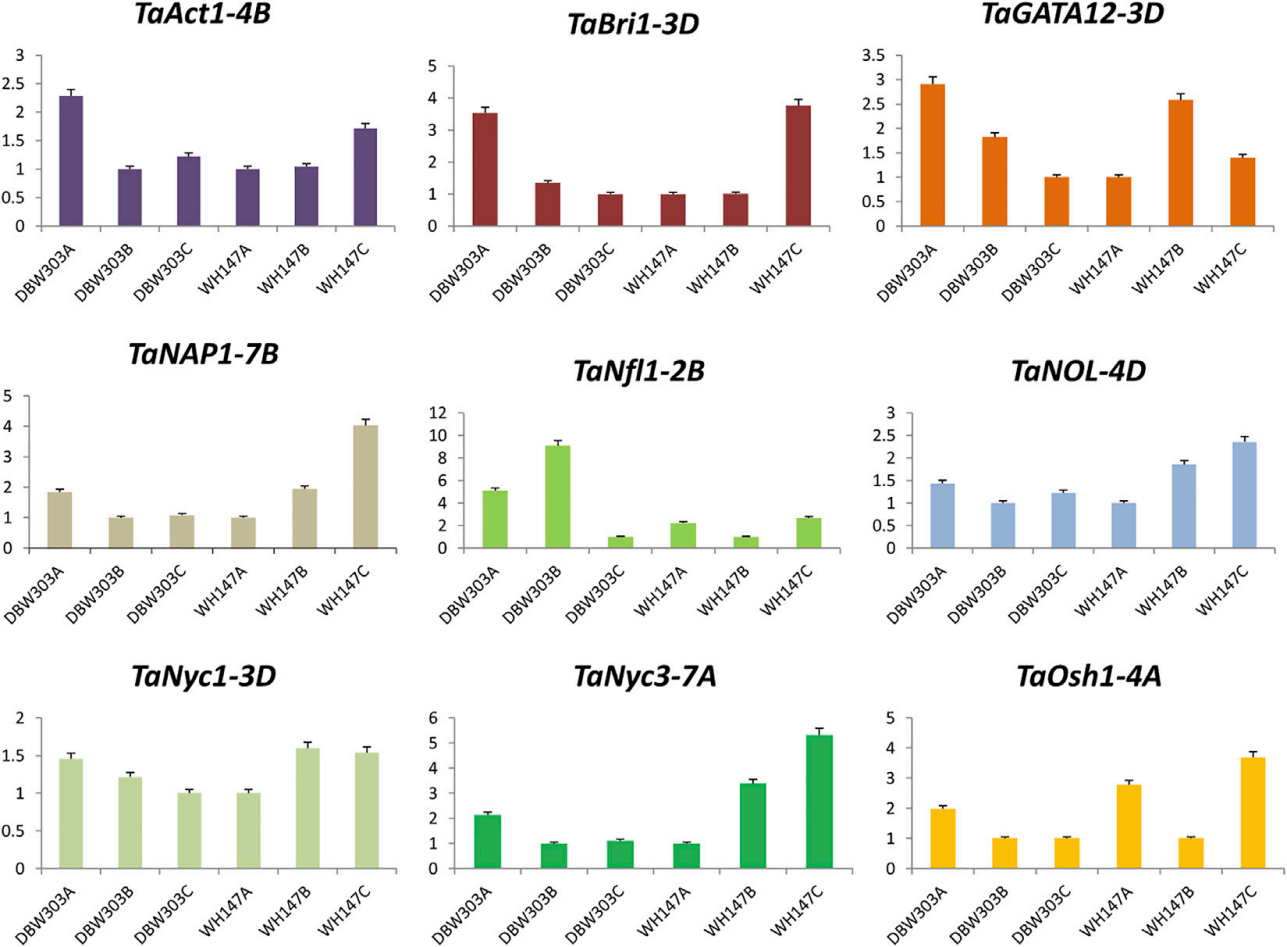
Graphical representation of relative expression levels of nine candidate genes in two wheat varieties namely DBW303 and WH147 at three different developmental stages namely flag leaf emergence (DBW303A, WH147A); fully developed flag leaf (DBW303B, WH147B) and flag leaf at the time of senescence (DBW303C, WH147C).

**FIGURE 12:**
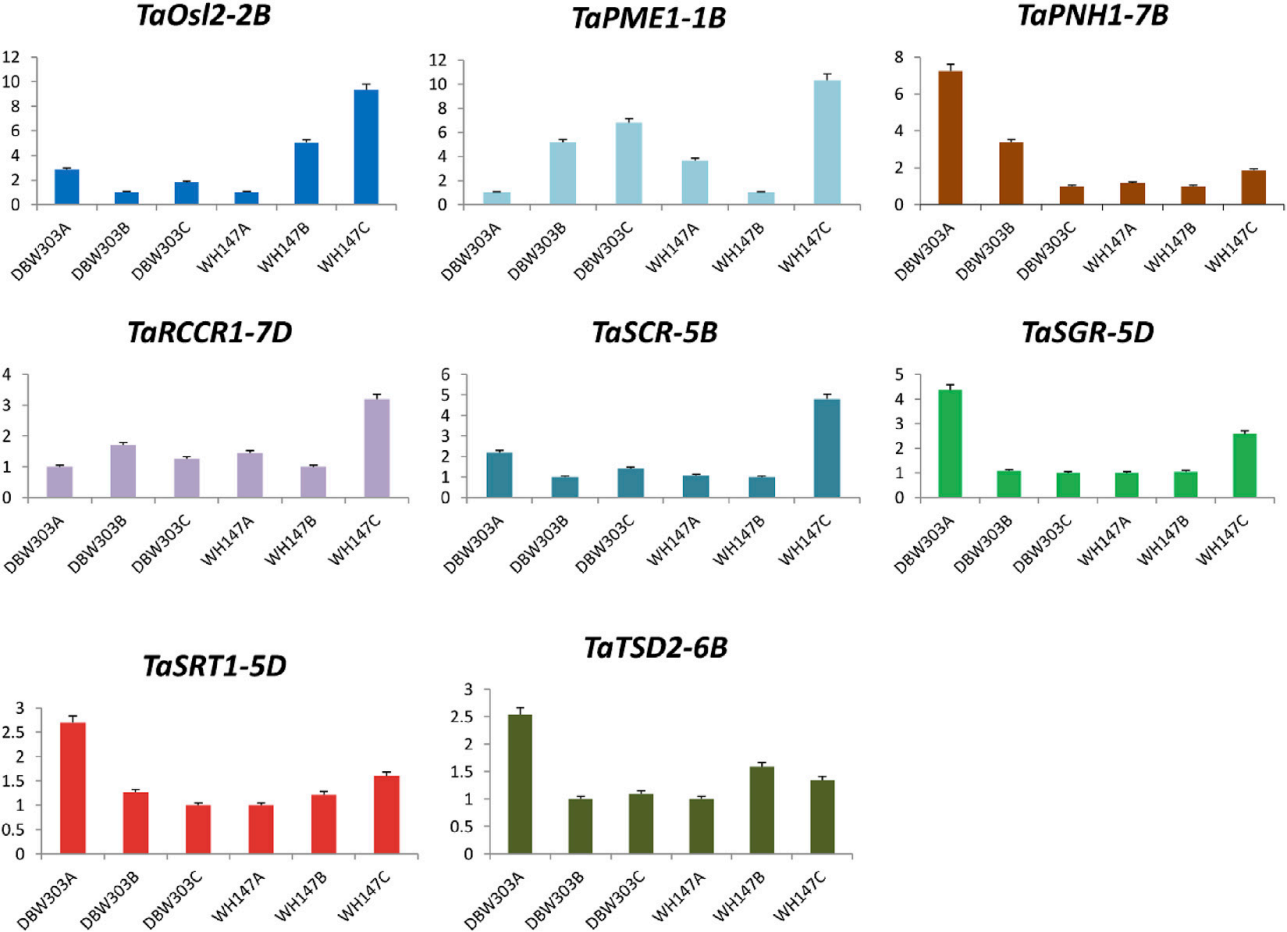
Graphical representation of relative expression levels of 8 candidate genes in two wheat varieties namely DBW303 and WH147 at three different developmental stages namely flag leaf emergence (DBW303A, WH147A); fully developed flag leaf (DBW303B, WH147B) and flag leaf at the time of senescence (DBW303C, WH147C) in the manuscript.

The expression of *TaAct-4B* was highest in the flag leaf emergence stage of the high-yielding wheat variety and was similar in *TaBri-3D*, *TaNfl1-2B*, *TaSGR-5D*, *TaSRT1-5D*, and *TaTSD2-6B genes*, indicating their constructive roles in the growth of flag leaf. The fold expression of genes such as *TaNyc1-3D*, *TaNyc3-7A*, *TaSCR-5B*, and *TaOsl2-2B* was more prominent during the senescence of flag leaf, revealing their important roles in senescence-related processes. Not just this expression level of these senescence-related genes is much higher in low-yielding wheat variety, that is, WH147 than the high-yield wheat variety DBW303 (Figures 11, 12).

### 3.10 Complex regulatory networks of flag leaf development and associated proteins

#### 3.10.1 Network of protein–protein interactions

Due to the unavailability of data for the wheat interactome in GeneMANIA, an interactome study was performed with reference to Arabidopsis. These putative interactor proteins were predicted at the *in silico* level to have regulatory-associated proteins based on physical interactions, shared functional domains, and co-expression. Of 17 proteins, interactome data for nine proteins were available in Arabidopsis. GeneMANIA (Franz et al., 2018) interactome analysis revealed interactions with the 20 most closely related proteins for each gene, allowing the elucidation of different functions based on these interactions.

TaBri1-3D has been discovered to interact with BAK1, CPI1, BSK1, BIK1, CRT3, SERK4, and 14 other proteins. The green lines show their interaction at the genetic level. The interactome analysis of TaBri1-3D revealed that this protein is involved in steroid metabolism, specifically the brassinosteroid-mediated signaling pathway, which is responsible for the negative regulation of cell death in plants. TaGATA12-3D was found to interact with 20 proteins of the same GATA family, including GATA18, GATA4, GATA5, and GATA2, which are basically involved in the circadian rhythm of plants. TaNyc1-3D was found to interact with NOL, HCAR, SGR1, SGRL, RCCR, and 15 other genes that were found to be involved in chlorophyll metabolic processes and nitrogen-containing compound catabolic processes. TaSRT1-5D was found to interact with SRT2, ETFA, HDA15, NUP62, GLDP1, PHB, DHS, ALS, HACL, PDC, CCA, and nine more genes. These were found to be involved in carboxy-lyase activity, the oxidoreductase complex, mitochondrial respiratory chain complex I, the NADH dehydrogenase complex, and the negative regulation of nitrogen compound metabolic processes. TaOsh1-4A was found to interact with a group of 20 genes, such as GILT, SHM, RHM, ETFB, and ETFQO involved in functions like hydrolase activity, nucleotide biosynthetic processes, and oxidoreductase activity acting on the CH-OH group of donors, NNAD, or NADP as acceptors. TaSGR-5D was found to interact with proteins such as SGRL, NOL, PPH, RCCR, HO1, NYC1, and HCAR, which were found to be involved in chlorophyll metabolic processes, mainly catabolic ones. TaNAP1-7B was found to interact with 20 proteins, including BRK1, FLP, ABIL1, MYB88, PNM1, and HDT2. These proteins were found to be involved in the positive regulation of protein polymerization, cellular component morphogenesis, and cellular component organization. TaSCR-5B was found to be involved in asymmetric cell division by interacting with proteins such as SCL, GASA, PER32, SHR, and different SCL elements. TaRCCR1-7D was found to interact with 20 proteins, including ALB, NYC, RVE, SGRL, NOL, PPH, and PAO. These were found to be involved in chlorophyll metabolic processes and cellular nitrogen compound catabolic processes (Supplementary Figure S2).

These proteins were selected together for interactome analysis, and two different clusters were found, one with Nyc1, NOL, SGR, and RCCR, which are involved in chlorophyll metabolic processes, and the second cluster includes NAP, Bri1, and SCR, which are involved in cellular organization and brassinosteroid-mediated cell signaling.

#### 3.10.2 Networks of chemical–protein interactions

The chemical–protein interaction analysis was performed with the help of the STITCH v5.0 server against *Hordeum vulgare* (Kuhn et al., 2007). The protein sequence of TaBri1-3D was found to interact with manganese and MgATP; TaGATA12-3D with MgATP, nitrate, ammonia, nitrite, and cycGMP; TaNOL-4D with nicotinamine, diphosphate, and reduced nitric acid; TaNyc1-3D with nicotinamine, reduced nitric acid, and TPNH; TaNyc3-7A with red chlorophyll, benzoic acid, sphinganine, and 7-keto-8-amine; TaPME1-1B with methanol, pectate, and distilled water; TaRCCR1-7D with red chlorophyll, hydrogen, sodium, TPNH, nicotinamine e and p, and SeMet; TaSCR-5B with R-rolipram and 1,2 dibromo 1; TaSGR-5D with CDCs, DMFE, and ketoglutarate; TaSRT1-5D with magnesium, vitamin B, carbamoyl phosphate, citrulline, ketoglutarate, hydrogen, ammonia, and phosphate; and TaTSD2-6B with mifepristone, trichlorobiphenyl, and Pentachlorobiphenyl (Supplementary Figure S3).

After the analysis of interactions, it was quite evident that most of the proteins were found to be associated with various N-containing compounds and enzymes related to nitrogen metabolism. Therefore, the importance of nitrogen and chlorophyll metabolism in flag leaf development was quite evident from the predicted results.

Nitrogen-use efficiency depends on nitrogen uptake, assimilation, and remobilization. In cereal crops such as wheat, mature leaves work as a sink to store nitrogen before the stage of anthesis, and upon anthesis, these leaves serve as the source of nitrogen to support the process of grain filling. Plants absorb N in their inorganic forms such as nitrate and ammonia, most of which are assimilated into organic forms. Mature tissue stores all organic and unassimilated inorganic nitrogen, either directly or indirectly utilized by the expanding tissue.

### 3.11 Molecular dynamics simulation of predicted proteins

**FIGURE 13:**
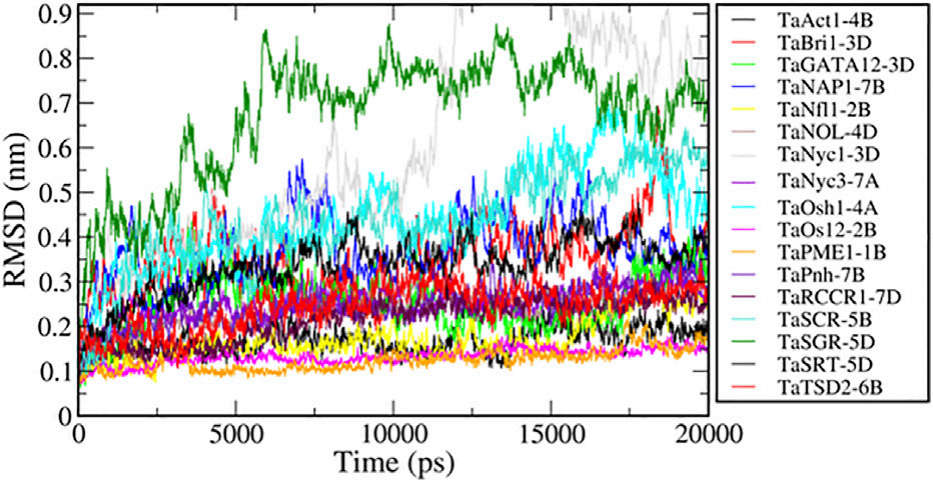
MD simulations of genes associated with flag leaf development.

Molecular dynamics simulations have been extensively used to explore the conformational behavior of proteins. Here, we performed 20-nanosecond MD simulation studies of seventeen modeled flag leaf genes to understand their structural behavior. To investigate the equilibration and protein stability during the simulations of these proteins, the Cα RMSDs were calculated and monitored over the course of 20-nanosecond simulations. The assessment of the structural change was carried out by the analysis of the RMSDs from the starting structures as a function of simulation time. The resulting RMSD plots, presenting the conformational changes during simulation, showed that all the proteins (except two) achieved equilibrium at ∼5 ns and remained stable for a period of 20 ns (Figure 13). These analyses also suggest that there are no large structural changes observed during simulation.

The RMSD values of two proteins, TaNyc1-D and TaSGR-5D, show higher values (>0.6 nm), indicating that these proteins have large conformational changes during the simulation. We used principal component analysis to better understand conformational changes during the simulation. PCA is a widely used method to reveal concerted motions (fluctuations) with large amplitudes (structural variations) from a set of configurations (Hess et al., 2008). All the simulated trajectories were projected along with the first principal component (PC1) which represents the largest structural variations. The motions along PC1 for all the trajectories were rendered (Supplementary Figure S4). Motions along PC1 showed that the large conformational changes in TaNyc1-D were due to the relative movement of different subunits, and the intra subunit conformational changes were not significant in TaSGR-5D due to the movement of flexible loops present at the terminal regions.

### 3.12 Phylogenetic analysis

**FIGURE 14:**
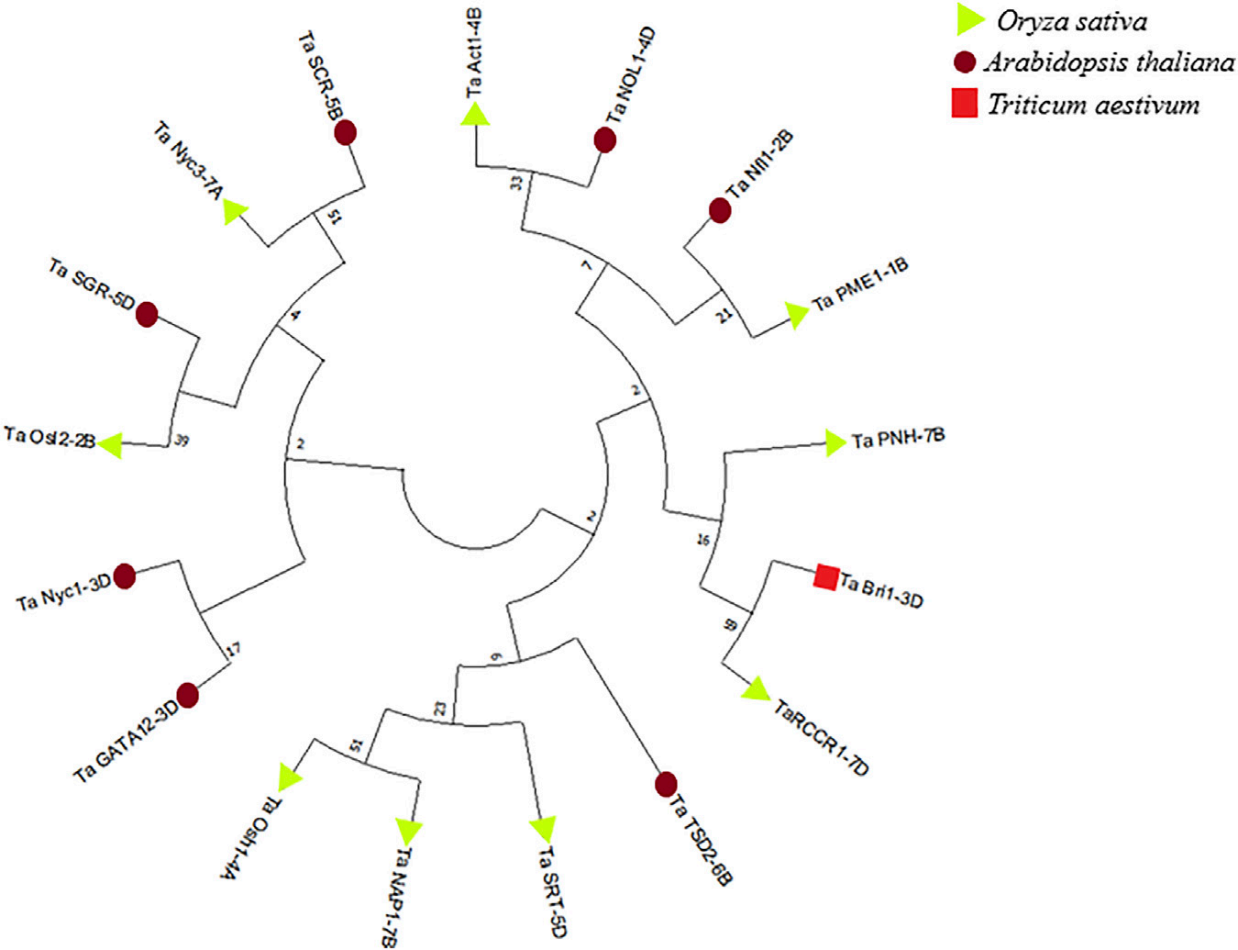
Phylogenetic tree of proteins associated with the development of flag leaf constructed through MegaX software.

Phylogenetic analysis was performed by using sequences of predicted proteins associated with flag leaf development through Mega-X software (Kumar S. et al., 2018). A phylogenetic tree was constructed using the neighbor-joining method with 1,000 bootstraps for inferring evolutionary relationships. Two distinct clusters were obtained, one with the genes involved in developmental processes and the other with the genes involved in degradation processes such as chlorophyll degradation and cell wall degradation. As inferred from Figure 14, the genes involved in the developmental processes made up a larger cluster than those in the other cluster.

## 4 Discussion

All 17 candidate genes associated with flag leaf development in wheat were characterized with the help of various bioinformatics tools. Phylogenetic analysis was the most prominent basis for the classification of these genes, under two distinct clusters, one with most of the genes associated with development and the other with genes associated with the regulation of senescence in flag leaf.

The first cluster included development-associated genes such as *TaAct1-4B*, *TaNfl1-2B*, *TaPME1-1B*, *TaPNH1-7B*, *TaBri1-3D*, *TaRCCR1-7D*, *TaTSD2-6B*, *TaSRT-5D*, and *TaOsh1-4A*, except *TaNAP1-7B* and *TaNOL1-4D*, whereas the second cluster included senescence-associated genes such as *TaSCR-5B*, *TaNyc3-7A*, *TaSGR-5D*, *TaOsl2-2B*, *TaNyc1-3D*, and *TaGATA12-3D*. Overall, starting from the functional enrichment analysis followed by the expression analysis, interaction studies, and structure-based functional analysis, this classification of genes fitted the best in understanding the whole picture of flag leaf development to a larger extent. The role of these genes as individuals or in collaboration with other genes was too complex to distinctively group them; hence, the genes are discussed in accordance with phylogenetic clusters.

TaAct1-4B, a member of the NBD-sugar kinase-Hsp70_act superfamily, plays an important role in cytoskeletal organization, localization, and transport of photoassimilates. Gui et al. (2015) reported that plasmodesmata conductance for the transport of photoassimilates in rice is regulated by the interaction of grain setting defect 1 with *OsACT1* (Gui et al., 2015). Moreover, it was the most prominent gene that was found to be expressed in the flag leaf of wheat, as was deciphered through *in silico* gene expression analysis and was the most prominent during the flag leaf emergence stage in the high-yielding wheat variety. Nfl in tobacco was found to specify determinacy for both flowers and leaves in progenitor cells as Nfl was found to be expressed in vegetative tissues (Hofer et al., 1997). TaNfl1-2B was found to be a member of the C-LFY-FLO superfamily, expressed predominantly during stages such as inflorescence emergence and germination as studied through gene ontology and expression analysis. The highest amount of TaNfl1-2B was found during the developmental stages of flag leaf in the high-yielding wheat variety. Similar results were revealed by Ahearn et al. (2001) signifying the critical role of Nfl1 in the allocation of meristematic cells that are responsible for the differentiation of lateral structures such as leaves and branches (Ahearn et al., 2001).

TaPME1-1B was found to be a pectinesterase, and its interaction with pectate was confirmed through the STITCH-based chemical protein analysis. Pectin methylesterase (PME) is a carbohydrate esterase family member that cleaves the ester bond between a methyl group and galacturonic acid. The optimal pectin methyl esterification in each cell type is determined by the balance between PME activity and PME inhibitors regulated by posttranslational PME inhibition. The overexpression of PMEI28 was found to result in reduced culm diameter and dwarf phenotypes in transgenic rice plants. It was found to function as a critical structure modulator by regulating the degree of pectin methyl esterification. Impairment in pectin methyl esterification affects physiological properties of the cell wall components and causes abnormal cell extensibility in culm tissue (Nguyen et al., 2017).

TaPNH1-7B, an Argonaute (PAZ Piwi domain) protein, found in the cytoplasm and intracellular spaces, has nuclease activity and acts as a positive regulator of leaf development, as evident through its predominant expression in the apex and axillary bud in *in silico* analysis. The fold expression of *TaPNH1-7B* was found to be highest during flag leaf emergence followed by fully grown flag leaf stages in the high-yielding wheat variety. The expression of *TaPNH1-7B* was optimally lower during all stages of flag leaf development in the low-yielding wheat variety, so it can be a potent gene to work with to increase the wheat yield. It was found by Nishimura et al. (2002) that OsPnh not only functions in S-adenosyl methionine (SAM) maintenance but also in leaf formation directly through vascular development. Malformed leaves with abnormal internal vascular structure were observed in antisense *OsPnh1* plants (Nishimura et al., 2002). TaBri1-3D, an LRR from the ribonuclease inhibitor-like superfamily, was found to positively regulate the development of the flag leaf in wheat. Decreased plant height with compact stature, narrow and short leaves, short internodes, decreased brassinosteroid response, and expression of BR-related genes were reported in *Brachypodium distachyon* with the BRI1-RNAi mutation (Feng et al., 2015). This supports the increased expression of *TaBri1-3D* during flag leaf development. Although most researchers primarily stress the influence of brassinosteroid-signaling genes on factors such as plant height, the impact on leaf architecture is also apparent. Altered leaf architecture, along with other brassinosteroid-insensitive phenotypic traits, was shown in Uzu 1, a mutant of barley with altered signaling of brassinosteroid due to the exchange of amino acids in the *HvBRI1* gene (Dockter et al., 2014). Similarly, shorter leaf blades and sheaths after the knockdown of *BRI1* homologs were found in maize plants (Kir et al., 2015). In ryegrass genotypes, the deletion in the LpBRI1 locus resulted in significantly narrower leaves than that of the genotypes in which the deletion was harbored (Statkevičiūtė et al., 2018). Chemical network analysis of TaBri1-D revealed its involvement in carboxylase and nitrate domains, both for synthase and transferase. A dwarf and low tillering (dlt) mutant of rice was characterized, and cloning of the dlt gene was performed through map-based cloning. DLT was found to encode a new member of the plant-specific GRAS family. The dwarf phenotype of *dlt* is similar to that of the BR-deficient mutant of rice (Tong et al., 2009).

TaRCCR1-7D was found to be a red chlorophyll catabolite reductase. Through microarray analysis, it was found to be predominantly present during developmental stages such as germination, stem elongation, and booting. The *in silico* expression of *TaRCCR1-7D* during the development of the flag leaf was found to be highly increased compared to that of other genes. In contrast, Sakuraba et al. (2013) reported less-pronounced changes in RCCR in developing or senescing leaves (Sakuraba et al., 2013). Through PPI and PCI network analyses, TaRCCR1-7D was found to interact with red chlorophyll and genes responsible for its degradation, such as *Nyc, SGRL*, and *NOL*, indicating its potential role in the reduction of chlorophyll catabolic genes and indirectly helping in the developmental processes.

*TaTSD2-6B* expression, which is a member of the methyltransferase 29 family, was found to be increased during the emergence of the flag leaf and unaffected during the senescence stage when analyzed through GENEVESTIGATOR. Similar results were found through expression analysis in high-yielding wheat varieties. Through chemical network analysis, TaTSD2-6B was found to interact with reduced cell adhesion and noncordial shoot development, similar to what was observed in the mutant of the TSD2 gene (Krupková et al., 2007).

TaSRT-5D, an inter-alpha trypsin inhibitor, was involved in stem elongation, and an increase in its expression during flag leaf emergence was also elucidated through a microarray analysis. TaSRT1-5D was found to be the most prominent in the high-yielding wheat variety during flag leaf emergence. Through network analysis, it was found to interact with intermediates of different metabolic cycles, such as the citric acid cycle. *OsSRT1*, one of the homologs of silent information regulator 2 (*SIR2*) in rice, was found to negatively regulate the process of leaf senescence by repressing the expression of the biosynthetic genes of the leaf senescence metabolic cascade and partially through H3K9 deacetylation of *OsPME1* (Fang et al., 2016). TaOsh1-4A, a homeobox family member, is highly important for the development of the plant. An Osh15 mutant revealed the importance of the *KNOX* gene in the self-regulation of other genes and SAM (Tsuda et al., 2011).

Of all the genes in cluster 1 discussed before, *TaNAP1-7B* and *TaNOL1-4D* were the only exceptions that were found to be not associated with developmental processes. The expression of *TaNAP1-7B* increased during the emergence stage of flag leaf development. It was found to be involved in cellular component morphogenesis and organization. OsNAP was found to act as an important link between ABA and leaf senescence in rice. The mutant named ps1, that is, a gain of function mutant, significantly exhibited premature senescence, in which the expression of ABA biosynthesis genes was found to be affected. The knockdown of OsNAP was found to produce an obvious delay in the process of senescence, which most importantly slowed the decrease in the functional photosynthetic capacity and highly influenced the seed setting ratio to a higher extent (Liang et al., 2014). The expression analysis of TaNAP1-7B deciphered its negative role in stay-green character in wheat, as it was found to be highest during the flag leaf senescence stage of the low-yielding variety and lowest during that in the high-yielding variety. TaNOL1-4D was found to be a member of the SDR family, whose expression most likely increased during flag leaf senescence (Kusaba et al., 2007; Park et al., 2007). Through network analysis, it was found to play an important role in carboxylic acid synthesis. When analyzed using a microarray, its increased level of expression during senescence was quite evident through its increased level of expression during senescence. *TaNOL-4D* was also found to be highly expressed in the low-yield wheat variety during flag leaf senescence; hence, its role in senescence is quite clear. The *Nyc3* mutant was found to retain more chlorophyll a and b content than the wild-type, and a significant decrease in senescence parameters during dark incubation suggested that it is a nonfunctional stay-green mutant. In addition, a small amount of chlorophyll a, pheophytin a, and Mg derivative without ^2+^ in its tetrapyrrole ring accumulated in the senescent leaves of the nyc3 mutant (Sato et al., 2009). Similarly, its expression was highest during the flag leaf senescence stage in the low-yield wheat variety.

Regardless of decrease in leaf functionality, the retention of chlorophyll during leaf senescence was increased in the mutant of the stay-green *SGR* gene in rice and its orthologs in *A. thaliana* (nonyellowing), *Festuca pratensis* (senescence-induced deficiency), tomato (green-fresh), pepper (chlorophyll retainer, and pea (I; known as Mendel’s green cotyledon gene) (Armstead et al., 2006; Jiang et al., 2007; Kusaba et al., 2007; Park et al., 2007; Aubry et al., 2008; Barry et al., 2008). *SGR* overexpression in rice seedlings resulted in the generation of singlet oxygen and activation of other reactive oxygen species and resulted in a chlorophyll-dependent regional cell death phenotype in the leaves (Jiang et al., 2011). It was evident that *OsSGR*, a member of the stay-green superfamily, plays an important role in the regulation of chlorophyll degradation, and the changes in the transcript expression levels of *OsSGR* are reflected in the regulation of jasmonic acid and cytokinin on chlorophyll degradation and leaf senescence. It was found to catalyze a key conversion from a red chlorophyll catabolite to a primary fluorescent catabolite during the degradation of chlorophyll (Liu et al., 2016). Chlorophyll was found to be degraded through the induction of light-harvesting chlorophyll a/b protein complex II (LHCPII) disassembly leading to the degradation of chlorophyll and chlorophyll-free LHCPII by proteases and catabolic enzymes, respectively (Park et al., 2007). However, the expression was highest in the flag leaf emergence stage in the high-yield wheat variety and flag leaf senescence stages in the low-yield wheat variety, depicting its contrary nature. TaOsl2-2B, an aspartate aminotransferase, was deciphered from network analysis and gene ontology analysis as being connected to various nitrogen-containing compounds. The overexpressed *OsL2* fusion protein in recombinant *E. coli* showed pyruvate-dependent-aminobutyric acid (GABA) transaminase activity. Induced *Osl2*-specific transcripts were found to be induced in the leaves that were senescing, and the chronological profile of accumulation of Osl2 protein was found to be linked with that of pyruvate-dependent GABA transaminase activity in the rice leaf (Ansari et al., 2005). The expression of TaOsl2-2B was also found to be elevated during the senescence of flag leaf, specifically in the low-yield wheat variety, as well as through microarray analysis indicating its significance as a senescence-associated gene.

TaNyc1-3D was found to interact with senescence-associated genes such as HCAR, SGR, and various nitrogen metabolites. Its expression was also prominently increased during flag leaf senescence. *Nyc1* encodes a membrane-localized short-chain dehydrogenase/reductase (SDR) that represents a chlorophyll b reductase that is required to catalyze the initial step of chlorophyll degradation. The *Nyc1* mutant, when analyzed, showed stay-green characters phenotypically. Similar stay-green phenotypes were reported in rice nol mutant such as *nyc1* mutants, that is, confirming that chlorophyll b degradation was selectively retained during senescence, resulting in the retention of thylakoid grana even at a later stage of senescence. It was found to severely inhibit the light-harvesting complex (Sato et al., 2009).

When over*expressed* in a semi-dwarf rice variety, *OsGATA12,* a zinc-finger transcription factor, caused an increase in the greenness of the leaf and a reduction in the tiller number and other yield-related attributes. The transgenic plants were found to be comparatively distinct from the wild type due to a significant increase in yield per area and harvest index as a result of reduced tillering. The increased greenness observed in the transformed plants was mostly due to decreased chlorophyll degradation along with chlorophyll synthesis, as the expression of genes involved in the degradation pathway of chlorophyll was also reduced (Lu et al., 2017). Therefore, TaGATA12-3D was found to regulate the processes of catabolism in plants, as revealed through the gene ontology analysis.

Functional enrichment analysis revealed the enzymatic functions of TaPME1-1B, TaNyc3-7A, TaNyc-3D1, TaNOL-4D, TaTSD2-6B, TaOsl2-2B, and TaRCCR1-7D and the regulatory functions of TaPnNH1-7B, TaBri1-3D, TaOsh1-4A, TaSRT1-5D, and TaAct1-4B in aiding cytoskeleton organization. Overexpression of genes associated with photosynthetic activity, nutrient transport indirectly maintaining greeness of plant tissue and downregulation or knocking out of the genes responsible for senescence associated activities like chlorophyll degradation and plant cell death. Both approaches can be given as individual trials or can be combined to obtain the best results regarding higher yields. It is quite evident from the results of MD simulations that all the candidate genes except *TaNyc1-3D* and *TaSGR-5D* were found to be stable under 20s RMAD analysis, and hence, they can be validated and used for further applications.

## 5 Conclusion and outlook for the future

With the concomitant development of genetic and molecular studies for the development of plants, a huge amount of understanding about the expression patterns of genes, their cellular locations, and molecular functions is necessary. It is obvious to realize that *in silico* approaches, involving the use of bioinformatics tools, remain cost-effective methods to make rapid analysis regarding the expected effect of genes; the more factors that are taken into account, the more accurate the prediction will be. Therefore, in this article, we performed the complete structural and functional annotation of genes related to different developmental stages of flag leaf so that direct or indirect higher grain yield can be achieved in the future by exploiting the information of these genes. Several genes associated with the development of flag leaf were identified and mapped to the genome. From the variations in characteristics from the structure of genes to microarray gene expression studies and phylogeny, it is quite evident that there is a great deal of complexity within the processes controlled by these genes. The 3D structures of proteins encoded by candidate genes were formed through a homology modeling-based approach. Modeled 3D structures of all the proteins were evaluated by using dihedral analysis of the Ramachandran plot. To assess the stability of modeled structures, molecular dynamics simulations after energy minimization were performed. By analyzing the results of both MD and homology modeling, the results were found to be consistent with the known set of investigational data. Based on our results, it has been concluded that these genes can be considered important candidates for the regulation of the development of the flag leaf in wheat. The thorough investigation of these genes associated with different processes such as leaf emergence, development, nutrient remobilization, and senescence indicated that the improvement in yield production can be attained by regulating the genes associated with the source–sink pathway and senescence of flag leaf. Leaf senescence is a major determinant of yield in many cereal crops. Stay-green, delayed senescence is associated with the retention of high-yield increments due to higher photosynthetic capacity during the active stage (Gentinetta et al., 1986; Thomas and Howarth, 2000). The stay-green sorghum variety B35 has been reported by Rosenow et al. (1983). It was found to show postflowering and drought resistance with a high contribution to stable and high-yield production (Rosenow, 1983). Through gene expression analysis, it was clear that by enhancing the expression of genes such as *TaSRT1-5D*, *TaPNH1-7B*, and *TaNfl1-2B* and by downregulating genes such as *TaNAP1-7B*, *TaNOL-4D*, and *TaOsl-2B*, high-yielding wheat varieties could be generated. Therefore, the data regarding the candidate genes can be utilized to manipulate these complex processes such as the source–sink pathway and senescence for the enhancement of yield in wheat. Despite comprehensive analysis in the current study, there is still much room for improvement before a complete array of regulation of these complex processes involved in flag leaf development through validating these genes through reverse genetics and biotechnological approaches.

## Data Availability

The original contributions presented in the study are included in the article/Supplementary Material; further inquiries can be directed to the corresponding author.

## Footnotes

1
www.agrochart.com

2 43bf642e28db4188a97b09ffb580409bd5e7a2a4 @www.fas.scot.
