# Supplementary material for: Structural and functional insights into the candidate genes associated with different developmental stages of flag leaf in bread wheat (Triticum aestivum L.)
Source: Front Genet. 2022 Aug 24;13:933560. doi: 10.3389/fgene.2022.933560 (PMC9449350; doi:10.3389/fgene.2022.933560)
Supplement: Supplementary file 1 [file DataSheet2.PDF]

Supplementary Table.1. List of primers for qRT-PCR based expression analysis

S No.	Name of the candidate gene	Primer Sequence	Amplicon length (bps)
1	*TaAct1-4B*	CCCCATCTACGAAGGATATGCT	150
		TTCACGTCCCTCACAATTTCC	
2	*TaBri1-3D*	CAAGGGCGACGTGTACAGCTA	150
		GGGTCAAACACATCGGTGATC	
3	*TaGATA12-3D*	TTCGTGATGTCGAGGCATTC	153
		CAGCAGGTGGCTCTCCATGT	
4	*TaNAP1-7B*	CTAGGCTGGCGAAGGAACAG	150
		GGACGATCCCAGCAGATAATTT	
5	*TaNfl1-2B*	GAAGAAGAACGGGCTGGACTAC	100
		AGACTTCTGGCCATGCAGCTT	
6	*TaNOL-4D*	TGGCATGGTTACGACTGATCTT	150
		GGCTTCATGGATTGGTTGGTA	
7	*TaNyc1-3D*	AATGTTGTCATAACGGGAAGCA	150
		GCCCTCCTGTATGTTCTCTTCAA	
8	*TaNyc3-7A*	CGCCAGCAGTGCTTTTTCTAC	150
		CCTTAGGAGCAGGGTCTTCACA	
9	*TaOsh1-4A*	AGATCGCTGCGCAATATCCTT	150
		GCTCAGGTACCCACTGTACTTCTTC	
10	*TaOsl2-2B*	GTGCCCATTGGAGCAACTCT	150
		CCGATAGATTTTCAACGCTTCA	
11	*TaPME1-1B*	TGCCCAAGAACAAGAAGTATGTCA	150
		GTCATATTCACCGCCACGAA	
12	*TaPNH1-7B*	TTGTCGGATGCAAACAGAATTAA	150
		TTGATCGTCGATTGGGAAGAT	
13		GAGCAGAGGAGGACACGAGAGA	150

	TaRCCR1-7D	CTGGATGGGCAGAGAAGGAA	
**14**	***TaSCR-5B***	ACAAGGCCGGGAATCTTGAC	128
		TTTGATGAGACACAGCGTGTTG	
**15**	***TaSGR-5D***	CGCTCCGCTACTACATCTTC	125
		GTGGAAGTAGACCCACACC	
**16**	***TaSRT1-5D***	AAACAAGCAACTGGAGCAAATG	150
		GGCTCCTCTGGTTTCTTTGGT	
**17**	***TaTSD2-6B***	TACGACCAGAGGGCTGGATTA	150
		GAAAGGGCTTCTGACAGACCAA	
**18**	***Actin***	AGTGGAGGTTCTACCATGTTTCCT	118
		CACTGTATTTCCTTTCAGGTGGTG	
