# Supplementary material for: Structural and functional insights into the candidate genes associated with different developmental stages of flag leaf in bread wheat (Triticum aestivum L.)
Source: Front Genet. 2022 Aug 24;13:933560. doi: 10.3389/fgene.2022.933560 (PMC9449350; doi:10.3389/fgene.2022.933560)
Supplement: Supplementary file 2 [file DataSheet1.PDF]

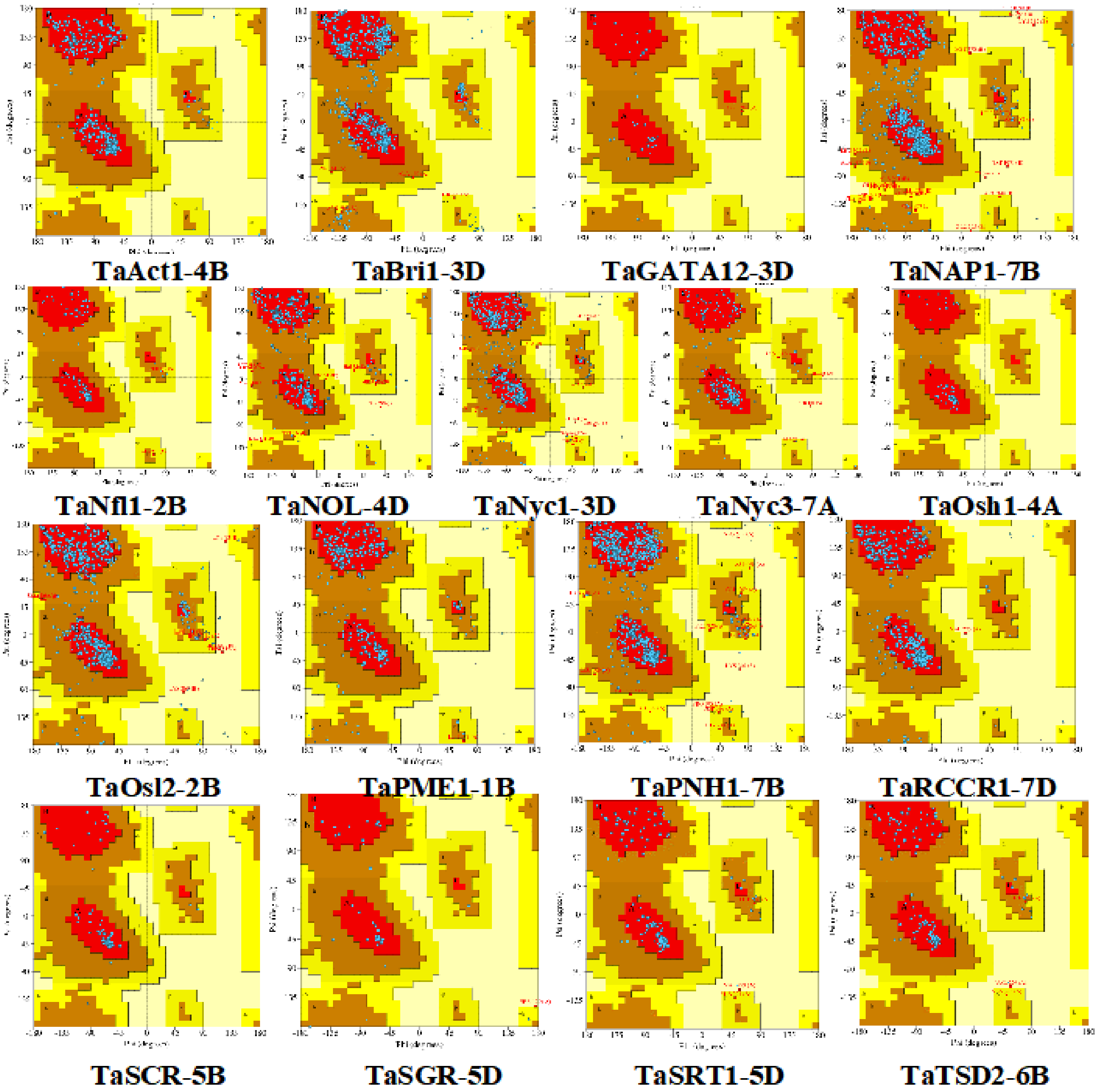

Figure S1. Ramachandran plots of proteins prepared for dihedral analysis through PROCHECK and SWISS-MODEL serve

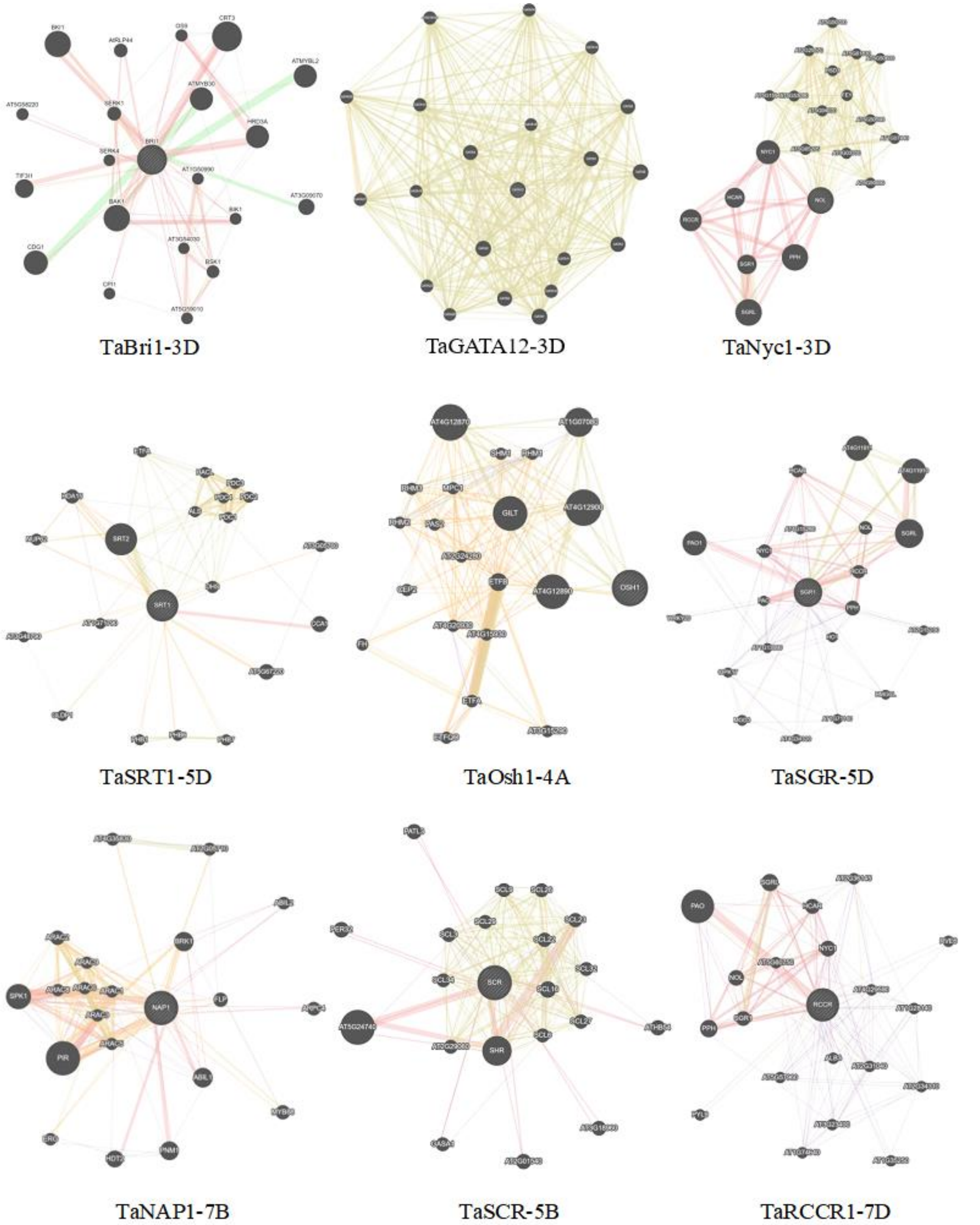

Figure S2. Regulatory networks of flag leaf development associated proteins and their interaction partners obtained through GeneMANIA

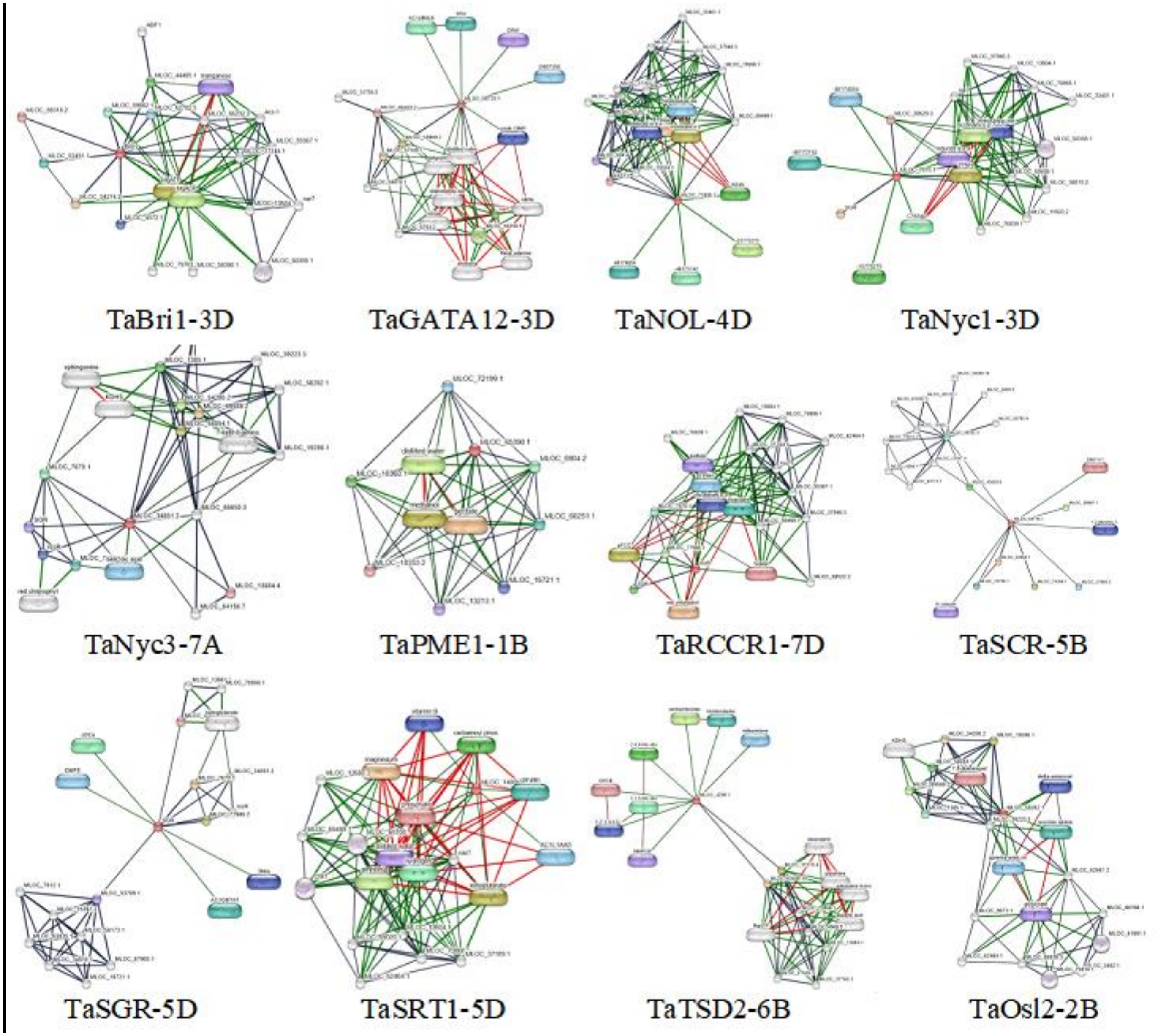

Figure S3. Predicted chemical partners of the identified genes through the STITCH v 5.0 server

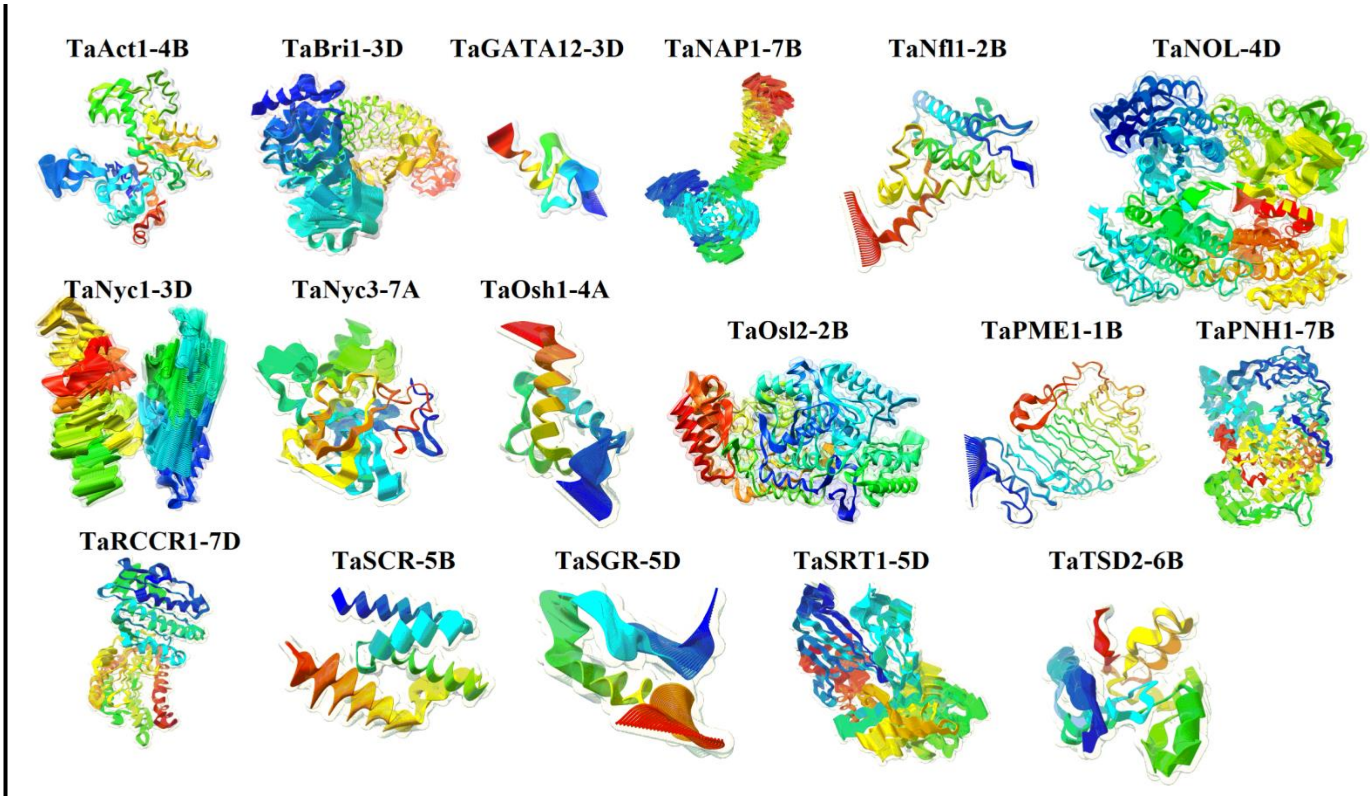

Figure S4. Graphical images of predicted proteins after MD simulation analysis of genes associated with flag leaf development.
